# Effects of a low-load multi-component training program with blood flow restriction versus the same program without blood flow restriction on muscle thickness and functional outcomes in physically inactive young adults: randomized controlled trial

**DOI:** 10.3389/fphys.2026.1792481

**Published:** 2026-04-01

**Authors:** Irfan Ahmad, Yali Feng, Jie Pu, Shaoqi Wen, Rui Zhong, Yifan Lv, Lehua Yu, An Zhang, Ying Yin, Botao Tan

**Affiliations:** 1 Department of Rehabilitation Medicine, 2nd Affiliated Hospital of Chongqing Medical University, Chongqing, China; 2 Department of Critical Care Medicine, 2^nd^ Hospital, Chongqing Medical University, Chongqing, China

**Keywords:** blood flow restriction, core strength, low-load multi-component training, physical fitness, sedentary behavior, students health

## Abstract

**Introduction:**

Low-load resistance training [≤50% one-repetition maximum (1-RM)] produces modest hypertrophic adaptations in untrained individuals. Blood flow restriction (BFR) training, combining low mechanical loads (30–40% 1-RM) with proximal limb occlusion, may augment these adaptations by inducing metabolic stress comparable to higher-load exercise. However, evidence comparing low-load multi-component training with and without BFR in physically inactive young adults remains limited. This study examined whether adding BFR to a standardized low-load training program enhances muscle thickness and functional performance outcomes compared with the same program performed without BFR.

**Methods:**

In this single-blind randomized controlled trial conducted at Chongqing Medical University from November 2024 to November 2025, we enrolled 48 physically inactive physically inactive university-aged adults (25 males; mean age 18.98 ± 0.64 years). Participants were randomly assigned (1:1) to receive either low-load multi-component with BFR (n=24) or low-load multi-component without BFR) (n=24) training for 6 weeks (4 sessions/week). The BFR group trained at 30% (weeks 1-3) to 40% (weeks 4-6) 1-RM, with pneumatic cuffs inflated to 50% of individual arterial occlusion pressure. Outcomes were muscle thickness (ultrasound), and physical fitness tests.

**Results:**

All 48 participants (mean age 19.0 ± 0.6 years) completed the 6-week intervention with ≥85% session attendance. The BFR group demonstrated significantly greater muscle thickness increases compared with without-BFR group in bilateral biceps brachii (right: +0.45 cm vs +0.11 cm, P = 0.001, ηp^2^=0.20; left: +0.37 cm vs +0.10 cm, P = 0.001, ηp^2^=0.21) and right rectus femoris (+0.13 cm vs +0.02 cm, P = 0.001, ηp^2^=0.17). Functional performance improvements favoring BFR included left-hand grip strength (+3.63 kg vs +1.09 kg, P = 0.001, ηp^2^=0.28), bilateral thigh circumference (P = 0.001, ηp^2^=0.12), and exercise-specific core training (males: pull-ups +5.92 vs +2.08 repetitions; females: abdominal curls +11.18 vs +3.33 repetitions). Between-group differences reached significance for 10 of 18 primary and secondary outcomes (56%). Seven BFR participants (29%) reported minor, transient discomfort during week 1; no serious adverse events occurred.

**Conclusion:**

Adding BFR to low-load multi-component training produced greater improvements in limb muscle thickness and functional performance compared with the same training performed without BFR in physically inactive young adults over 6 weeks. Benefits were most evident in upper extremity hypertrophy and task-specific functional capacity, with 56% of outcomes demonstrating significant between-group differences favoring BFR. The intervention was well-tolerated with no serious adverse events. These findings support BFR-enhanced low-load training as a potential alternative for individuals unable or unwilling to engage in high-load resistance training, though generalizability to other populations and longer-term sustainability require further investigation.

**Clinical Trial Registration:**

https://www.thaiclinicaltrials.org/, identifier TCTR20241110003.

## 1 Introduction

Resistance training is the primary intervention for enhancing muscular strength and hypertrophy in healthy individuals, with conventional guidelines suggesting training loads over 65% of one-repetition maximum (1-RM) to maximize muscle growth (American College of Sports Medicine, 2009). Although high-intensity resistance training has various practical and physiological barriers, such as significant joint stress, increased injury risk, prolonged recovery needs, and accessibility challenges associated with specialized equipment and supervision (Roberts et al., 2024) (Lavigne et al., 2025). These barriers are especially crucial for young adults, including physically inactive university-aged students, who may have limited access to extensive training facilities, face time pressures from curricular responsibilities, or have concerns regarding injury risk related to heavy-load resistance training.

Physical inactivity among university students is a rising public health issue, with recent studies revealing that significant percentages of this demographic do not adhere to prescribed physical activity requirements (Savage et al., 2024) (Barbosa et al., 2025). Patterns of sedentary behavior formed during university years frequently continue into later adulthood, leading to lifelong health impacts such as heightened risk of metabolic disorders, cardiovascular dysfunction, and gradual muscle mass reduction (Biddle et al., 2010). Providing effective, accessible, and safe resistance training alternatives for this demographic has major consequences for both short-term fitness results and long-term health outcomes.

Blood flow restriction (BFR) training, originating from the Japanese term Kaatsu training, has developed as an effective replacement to traditional high-load resistance training in the past twenty-five years (Patterson et al., 2019) (Loenneke et al., 2025) (de Queiros et al., 2025). This approach involves putting external pressure on the proximal segment of a leg during low-load resistance exercise (often 20-40% of one-repetition maximum), inducing localized hypoxia by partially restricting arterial inflow and more thoroughly obstructing venous outflow (Patterson et al., 2019) (Hayat et al., 2025). The resulting metabolic conditions—characterized by the accumulation of metabolites such as lactate, hydrogen ions, and inorganic phosphate, along with cellular edema and localized hypoxia—seems to contribute a significant impetus for muscle hypertrophy and strength enhancement, even with markedly decreased mechanical loading (Curovic, 2025) (Koloskov et al., 2025).

The physiological mechanisms responsible for BFR-induced muscle adaptations are complex and remain under investigation. Metabolic stress acts as a principal catalyst, with the development of metabolic waste inducing cellular swelling that stimulates the mammalian target of rapamycin (mTOR) signaling pathway, a vital regulator of muscle protein synthesis (Li et al., 2025) (He et al., 2025). The hypoxic conditions induced by BFR selectively activates higher-threshold motor units and type II muscle fibers, which are usually engaged during high-intensity contractions, thus delivering a neuromuscular stimulus similar to heavy-load training while utilizing significantly lighter weights (Iskarevskii et al., 2024) (Olmos et al., 2024). Systemic hormonal responses, such as increases in growth hormone and insulin-like growth factor-1, may enhance local anabolic signals, leading to extensive hypertrophic changes (Yinghao et al., 2021) (Aram et al., 2024).

Additionally, Chang et al. reported that BFR training (under proper conditions, i.e appropriate pressure, intermittent cuff inflation, and more frequent training sessions) yields muscle strength and hypertrophy improvements comparable to high-load resistance training in untrained individuals, whereas trained populations may experience even higher advantages from BFR regimens (Chang et al., 2024). Ma et al. also showed in their systematic review that BFR (20%–40% of 1 RM) along with resistance training yields muscle thickness and strength enhancements comparable to traditional training in young people (Ma et al., 2024). Bommasamudram et al. expanded upon these findings by indicating that BFR bodyweight resistance training provides potential for enhancing muscle growth and strength gains comparable to traditional methods, with certain studies indicating strength enhancements of 4–11% (Bommasamudram et al., 2025). While LL-BFR can elicit meaningful strength improvements, the broader evidence in healthy adults indicates that HL-RT typically produces larger gains in maximal strength than LL-BFR, whereas hypertrophy responses are often comparable (Lixandrão et al., 2018) (Wang et al., 2025). Accordingly, BFR should be viewed primarily as a pragmatic low-load strategy when high mechanical loading is undesirable or impractical, rather than a universal superior approach for maximal strength development (Lixandrão et al., 2018).

Despite a rapidly expanding evidence base, important gaps remain in the application of BFR training, particularly in young, healthy populations such as physically inactive university-aged adultss. Most BFR research has focused on clinical cohorts (e.g., post-surgical or older adults) or trained athletes, leaving limited evidence on their efficacy in physically inactive university-aged adults, a group that could substantially benefit from low-load, accessible training strategies. In addition, prior studies have often examined isolated muscles or single exercises, with comparatively few investigations evaluating whole-body BFR protocols, individualized AOP, specified adherence/safety, and clinically relevant functional outcomes that better reflect real-world training practice. Finally, potential gender-specific differences in adaptive responses to BFR remain poorly characterized, as many studies are male-dominant (Nancekievill et al., 2025) (Refalo et al., 2025).

Compared with traditional medium to high-load resistance training, BFR offers several practical advantages. The use of markedly lower external loads (typically 30–40% 1-RM vs ≥65% 1-RM) reduces mechanical stress on joints and may lower injury risk, which is particularly relevant for beginners and individuals with joint concerns (de Lemos Muller et al., 2024). Moreover, BFR requires minimal equipment and provides a novel training stimulus that may enhance accessibility and motivation. Importantly, accumulating evidence supports the safety of BFR when appropriately prescribed and supervised, with adverse event rates comparable to those reported for traditional resistance training (Patterson et al., 2019) (Jørgensen et al., 2016) (Feng et al., 2024) (Wang et al., 2022). Importantly, because the present trial compares LL-BFR against matched training without BFR, we also contextualize our work within studies directly evaluating this comparison. Meta-analytic evidence generally suggests LL-BFR produces greater strength and hypertrophy adaptations than traditional low-load resistance training alone, although the magnitude of benefit varies with training prescription and population (Loenneke et al., 2012b) (Fabero-Garrido et al., 2022).

Accordingly, this randomized controlled trial evaluated the effects of a 6-week low-load multi-component training with BFR versus low-load multi-component without-BFR in young adults (physically inactive university-aged). We hypothesized that low-intensity BFR exercise (30-40% 1-RM) would result in favorable gains in muscle thickness and functional performance. Using complementary outcomes, including ultrasound-derived muscle thickness, anthropometry, and performance tests, this study provides a comprehensive assessment of BFR efficacy in a population not well represented in the existing literature and contributes to evidence-based exercise prescription for young adults.

## 2 Methods

### 2.1 Study design and ethical approval

This study employed a two-arm, parallel-group, single-blind randomized controlled trial design to compare the efficacy of low-load multi-component training with BFR against low-load multi-component training without BFRin healthy physically inactive university-aged adults. The study protocol received approval from the Ethics Committee of Chongqing Medical University (approval number: 2024(55)) and was prospectively registered with the Thai Clinical Trial Registry (registration number: TCTR20241110003) before participant recruitment commenced. All procedures were conducted in accordance with the Declaration of Helsinki and reported according to the Consolidated Standards of Reporting Trials (CONSORT) 2010 guidelines.

Participant recruitment occurred between November 2024 to November 2025 through convenience sampling at Chongqing Medical University. Outcome assessors remained blind to all the study protocol just perform the pre-and post-assessment of all outcome measures.

### 2.2 Participants

#### 2.2.1 Sample size calculation

As the trial used a pre–post randomized design, the target effect was specified as the between-group difference in pre-to-post change (Δ) in the primary outcome. With two time points, this is equivalent to testing the group × time interaction. Therefore, the *a priori* calculation was used to estimate the sample size required to detect a clinically meaningful between-group difference in change. Based on an anticipated large between-group effect in change scores (standardized mean difference, d = 0.90) derived from prior BFR studies (Kang et al., 2015), the sample size was calculated for detecting a difference in change between groups (α=0.05, power=0.80). To account for a potential attrition rate of 10%, the required total sample size was calculated to be 48 participants, with 24 participants allocated to each group.

#### 2.2.2 Eligibility criteria

Inclusion criteria: Participants were eligible if they (1) were physically inactive university-aged adults aged 18–25 years at Chongqing Medical University; (2) were classified as physically inactive, defined as engaging in less than 150 minutes of moderate-intensity aerobic physical activity per week according to World Health Organization guidelines (Bull et al., 2004); (3) had no prior exposure to BFR training; and (4) provided written informed consent after receiving comprehensive explanation of study procedures and voluntary withdrawal rights.

Exclusion criteria: Participants were ineligible if they had (1) recent surgeries or musculoskeletal injuries within the past 6 months; (2) history of cardiovascular events or conditions contraindicated for exercise testing; (3) current use of hormonal medications or anabolic supplements; (4) tobacco use within the past 6 months; (5) any chronic health conditions that could interfere with exercise performance or safety; (6) current participation in structured resistance training programmes.

#### 2.2.3 Recruitment and randomization

**Figure 1:**
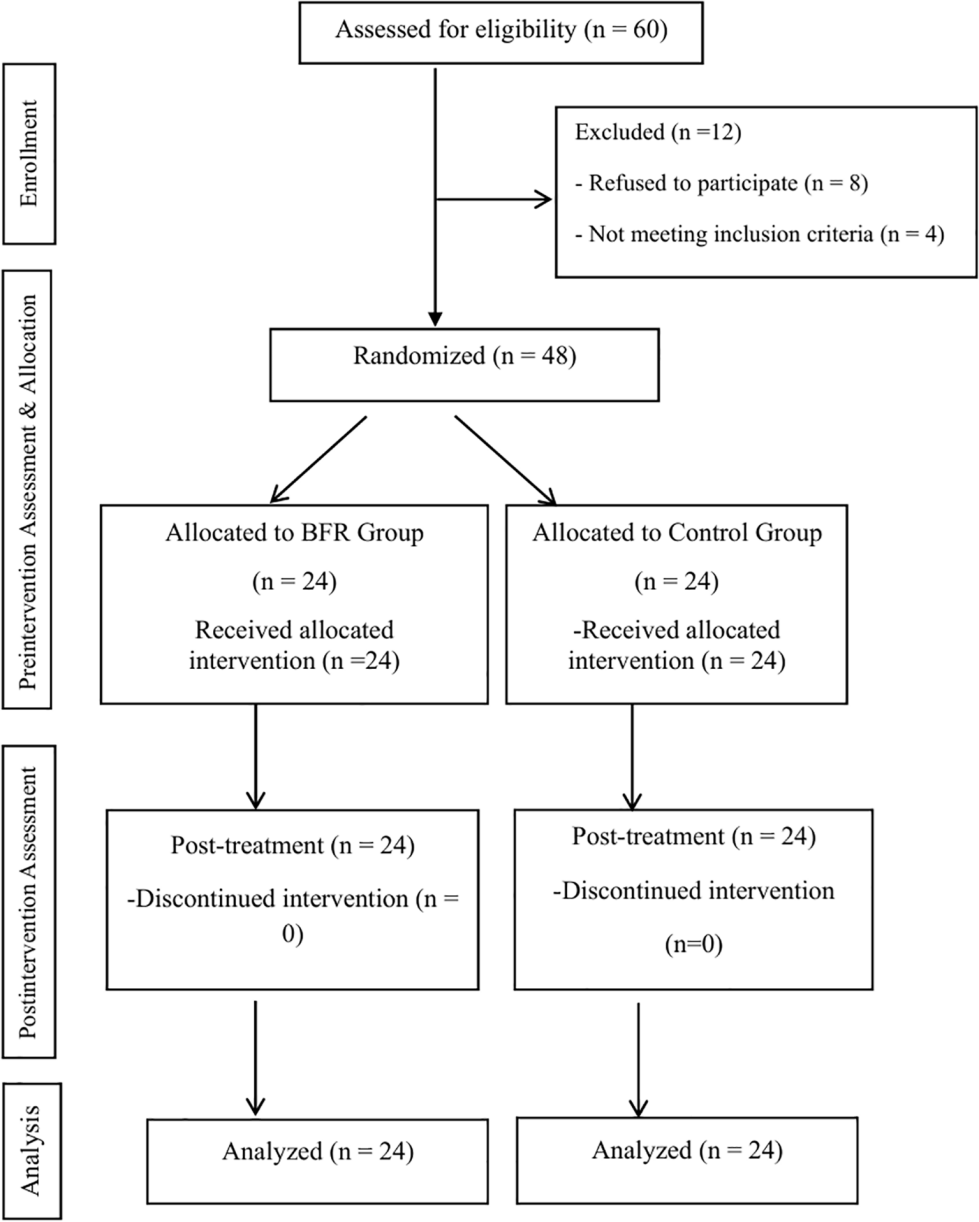
The CONSORT flow chart of recruitment and randomization of patients in the study.

Sixty physically inactive university-aged adults from diverse academic departments at Chongqing Medical University were initially screened for eligibility. Following screening, 48 participants (25 males, 23 females) met inclusion criteria and were enrolled. Randomization was performed using a computer-generated random sequence (L.P., S.W., R.Z.) with participants allocated in a 1:1 ratio to either the low-load multi-component training with BFR (n=24) or low-load multi-component training without BFR (n=24). Allocation concealment was maintained by storing the randomization sequence with a physiatrist (Yu.L.) who was not involved in data collection. Baseline characteristics (age, height, weight) were assessed for homogeneity between groups, with no significant differences detected (all p > 0.05), confirming successful randomization (**Figure 1**).

### 2.3 Interventions

#### 2.3.1 Low-load multi-component training with BFR protocol

**Figure 2:**
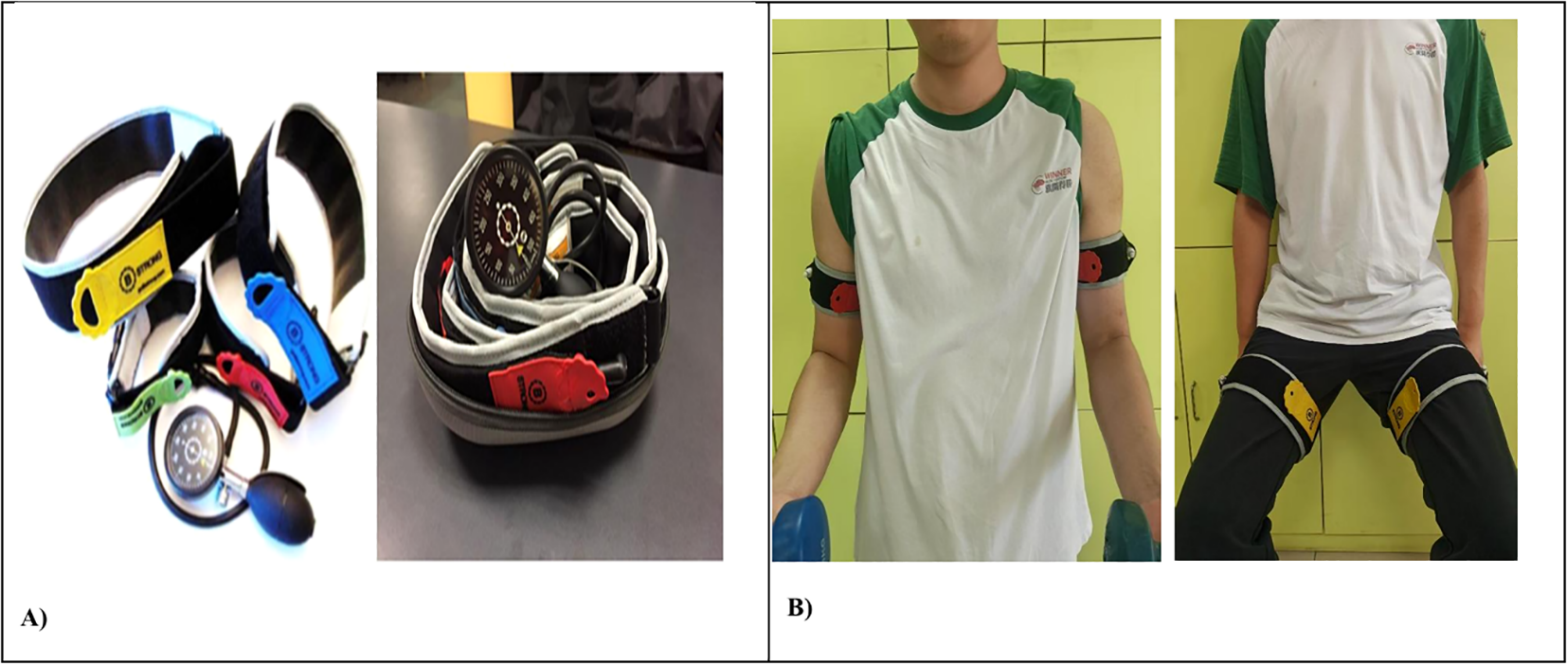
BFR Apparatus and Upper and lower limb application. **(A)** BFR Apparatus, **(B)** Lower and Upper limb application of BFR.

Participants assigned to the low-load multi-component training with BFR group performed resistance exercises with pneumatic cuffs (B-Strong Bands™, Stray Whales LLC, USA; 5 cm width for upper limbs, 7 cm width for lower limbs) applied to the proximal portions of exercising limbs. Cuffs were positioned at standardized anatomical landmarks: upper arm (at the deltoid-biceps junction) and proximal thigh (immediately inferior to the gluteal fold) **Figure 2**.

Individual arterial occlusion pressure (AOP) was determined for each participant before the intervention using Doppler ultrasound following validated protocols (Patterson et al., 2019). After 5 minutes of supine rest, pneumatic cuffs were inflated incrementally in 10-mmHg steps with 15-second intervals until complete arterial flow cessation was detected via Doppler ultrasound. Two measurements per limb were obtained and averaged to establish individual AOP values. Training occlusion pressure was set at 50% of individual AOP, resulting in pressures of 80–120 mmHg for upper limbs and 110–160 mmHg for lower limbs. Pressures were verified before each exercise set using calibrated digital manometers (accuracy ±2 mmHg), with real-time adjustments made if deviations exceeded 5% (Jessee et al., 2016) (Zhang et al., 2024).

Cuffs remained inflated during exercise sets but were deflated during rest periods between different exercises (for example, after completing all sets of arm flexion sets) to minimize cumulative ischemic exposure and enhance safety, consistent with current BFR guidelines (Hughes et al., 2017) (Loenneke et al., 2012a). Approximately 5 minutes of rest was provided when transitioning between different exercise types to allow for cuff pressure verification (BFR group) and partial metabolic recovery.

#### 2.3.2 Low-load multi-component training without BFR protocol

Participants in the low-load multi-component training without BFR group performed identical training under the same supervision, timing, and progression schedule as the BFR group, but without pneumatic cuff application.

### 2.4 Exercise training programme

Both groups completed a progressive, periodized 6-week training program comprising four supervised sessions per week (24 total sessions per participant). The training program delivered to both groups was a standardized, supervised, low-load, multi-component program that combined (i) speed/power elements (100-m sprint and weighted straight-leg jumps), (ii) low-load resistance and bodyweight exercises (e.g., squats, knee flexion/extension, upper-limb flexion/extension), and (iii) a core/upper-body endurance task (pull-ups or abdominal curl-ups). Thus, the study does not compare resistance training alone; rather, it compares the same combined program performed with versus without BFR applied during the resistance exercise component. Training was performed on alternate days, with one rest day between sessions to ensure adequate recovery. All training sessions were conducted at the Chongqing Medical University, under direct supervision of qualified physicians and physiatrists (A.Z., B.T., Y.Y.) and they remained present throughout all sessions to monitor for and manage any adverse events.

Each training session followed a standardized structure: (1) 5-minute warm-up consisting of full-body dynamic stretching exercises targeting major muscle groups (shoulder, elbow, hip, knee, ankle); (2) main training intervention (approximately 60 minutes); and (3) 5-minute cool-down involving static stretching and relaxation activities. **Table 1**.

**Table 1:** Training program.

Training program	Training content	Training time
Warm-ups	Subjects were instructed to perform a full-body warm-up in an organized manner. (Arm, forearm, hip, knee, ankle general stretching exercises)	Five minutes.
Main Intervention	100-meter sprint (once). Long Jumping: Shoulder sandbag (1.5-5kg) weight-bearing straight leg Jump 10 times (continuous jumping). Squats, Straight arm pull-ups (male), Abdominal curl (Female). Knee (flex/ext): knee movement range of 90° to 0°. Arm and forearm flex/ext), and hand exercise using mechanical hand grippers. 3 sets of each exercise, Weeks 1-3: 20 reps, Weeks 4-6: 30 reps, were performed with a 30 sec rest between sets but no rest between repetitions in each set.	Completion time per individual (~60 min).
Cool-down part	Subjects, perform full body relaxation activities. (Shoulder stretches, leg stretches, arm stretches, hip stretches, etc.)	Five minutes.

Exercises were performed in an order consistent with National Strength and Conditioning Association (NSCA) guidelines (Pearson et al., 2000) (Ratamess, 2017), which recommend sequencing movements from high-power activities to multi-joint strength exercises and finally single-joint exercises to minimize fatigue and preserve technique. Accordingly, each session began with explosive activities, starting with a single 100-m sprint, followed by weighted straight-leg jumps using a shoulder-loaded sandbag [1.5–3 kg (1–3 weeks), 4–5kg (4-6weeks]]. Participants then performed the primary multi-joint strength exercise for the lower limbs (squats), followed by the main upper-body or trunk exercise (straight-arm pull-ups for males or abdominal curls for females). Single-joint lower-limb exercises (knee flexion and extension through a 90°–0° range of motion) were performed next, and the session concluded with upper-limb assistance exercises, including arm and forearm flexion/extension and hand-grip exercises using mechanical grippers. All exercises were completed for three sets, with 20 repetitions during Weeks 1–3 and 30 repetitions during Weeks 4–6, separated by 30 sec of rest between sets, in line with NSCA principles prioritizing power exercises first, followed by large muscle-group movements and finally smaller muscle-group isolation work.

#### 2.4.1 Exercise-specific core training modifications

Participants performed different core exercises based on baseline functional capacity and safety considerations. Individuals able to safely execute straight-arm pull-ups with proper technique were assigned that exercise, whereas those unable to do so performed abdominal curl-ups as a lower-load alternative targeting the core musculature while minimizing upper-limb demands. This approach was adopted to ensure feasibility, reduce injury risk, and maintain an appropriate training stimulus for all participants. Although group allocation coincided with sex due to baseline strength differences observed during initial assessment, exercise selection was determined by performance capability rather than sex itself. Training volume and progression were standardized across exercise conditions to promote comparable relative intensity. Such individualized exercise prescription is consistent with established resistance-training principles, which recommend tailoring exercise modality to participant capacity to optimize safety, adherence, and physiological adaptation.

Training intensity and volume followed a progressive periodization scheme to prevent adaptation plateaus and optimize hypertrophic responses. Weeks 1–3: 30% of estimated 1-RM, 3 sets × 20 repetitions; Weeks 4–6: 40% of estimated 1-RM, 3 sets × 30 repetitions. 1–RM was calculated using submaximal repetitions-to-fatigue test using the Brzycki prediction equation (DiStasio, 2014).

Heart rate was monitored continuously during all training sessions using telemetry (Polar H10, Polar Electro Oy, Finland), with exercise intensity adjusted to maintain heart rate ≤85% of age-predicted maximum to standardize cardiovascular stress between groups.

### 2.5 Outcome measures

Outcome assessments were conducted at baseline and post-intervention by independent, blinded assessors who were not involved in the training sessions and had no access to group allocation or study hypotheses. The primary outcome measure (muscle thickness assessed by musculoskeletal ultrasound) was performed by a physician with formal training and extensive experience in musculoskeletal ultrasound imaging. Secondary outcome measures (including functional performance tests) were conducted by a qualified physiotherapist experienced in standardized outcome testing. Neither assessor was present at the intervention site to minimize potential assessment bias. All measurements were obtained in a standardized testing environment. Participants were instructed to avoid caffeine, alcohol, and strenuous physical activity for 24 hours before testing sessions, and to arrive in a fed but not satiated state (≥2 hours postprandial).

#### 2.5.1 Primary outcomes

##### 2.5.1.1 Muscle thickness (ultrasound imaging)

**Figure 3:**
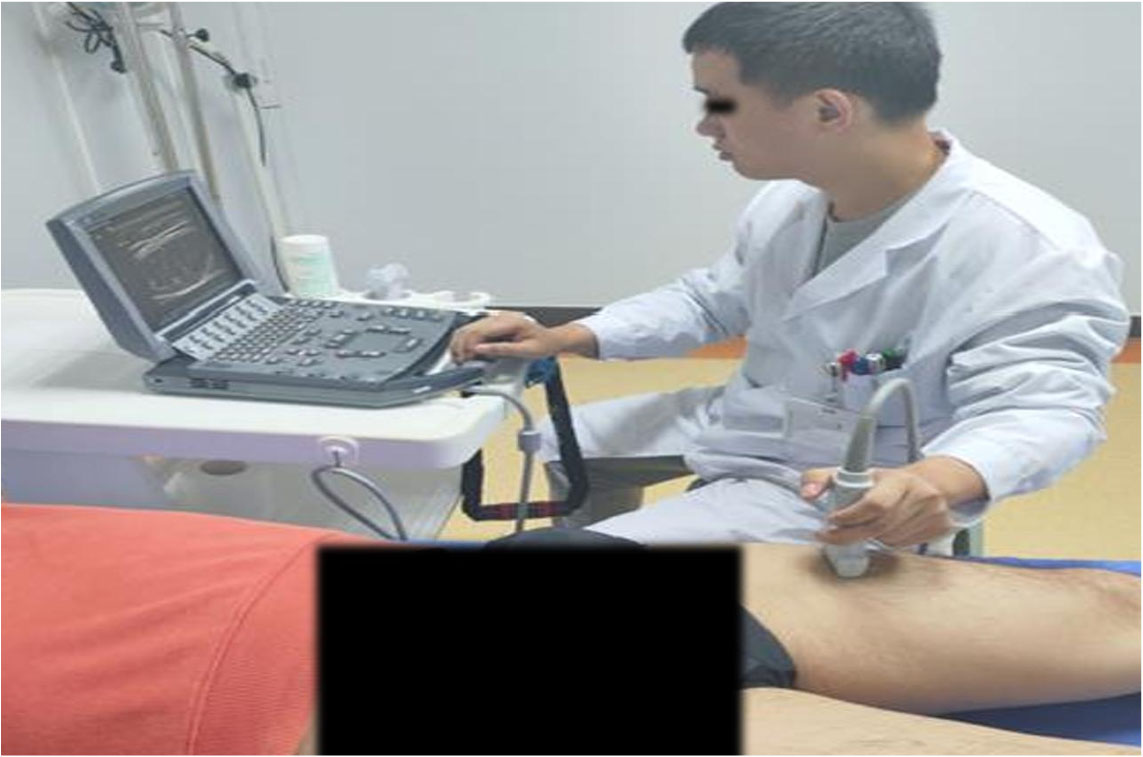
MSK ultrasound and its application.

Muscle thickness was quantified using B-mode ultrasound (Mindray DP-50, Mindray Medical International Ltd, Shenzhen, China) equipped with an 8.5-MHz linear array transducer (**Figure 3**). Ultrasound has demonstrated excellent reliability and validity for detecting training-induced changes in muscle morphology, with intraclass correlation coefficients exceeding 0.90 for experienced assessors (Högelin et al., 2022) (Lohmann et al., 2024).

##### 2.5.1.2 Measurement protocol

Participants were positioned in standardized postures for each muscle group: (1) biceps brachii-seated with elbow at 90°flexion, forearm resting on table; (2) rectus femoris-supine with feet straight; (3) gastrocnemius and soleus-prone with knee fully extended and ankle in neutral plantarflexion.

Measurement sites were identified as the midpoint between muscle origin and insertion on the most prominent aspect of the muscle belly, marked with semi-permanent ink to ensure identical positioning at post-intervention assessment. Skin was cleaned with 70% ethanol swabs before application of water-soluble transmission gel. The transducer was positioned perpendicular to the long axis of the muscle without applying compressive force to underlying tissue. Image acquisition parameters were standardized: gain = 50 dB, depth = 4.6 cm, frequency = 8.5 MHz.

Two images were captured for each measurement site, with the transducer lifted and repositioned between captures. Muscle thickness was defined as the perpendicular distance between superficial and deep aponeuroses (**Supplementary Figure 3B**), measured offline using integrated software calipers by an assessor blinded to group assignment and time point. The mean of two measurements was used for analysis, with a third measurement obtained if the initial two differed by >5%.

#### 2.5.2 Secondary outcomes

##### 2.5.2.1 Muscle circumference

Circumferential measurements of upper arm and thigh were obtained using a non-elastic measuring tape at standardized anatomical locations. Upper arm circumference was measured at the midpoint between acromion and olecranon with the elbow flexed to 90° and arm positioned at the participant’s side. Thigh circumference was measured at mid-thigh (mid distance from Anterior superior iliac spine to lateral epicondyle of femur) with the participant standing barefooted, knee in full extension, and weight distributed equally between limbs. Two measurements were obtained at each site. The mean value (recorded in cm) was used for analysis.

##### 2.5.2.2 Grip strength

Maximal isometric grip strength was assessed bilaterally using a calibrated handheld dynamometer (Baseline^®^ Hydraulic Hand Dynamometer, Fabrication Enterprises Inc., USA). Participants were seated with shoulders adducted, elbow flexed to 90°, and forearm in neutral position. They were instructed to squeeze the dynamometer with maximal effort for 5 seconds while receiving standardized verbal encouragement. Two trials were performed for each hand with 30-second rest intervals, and the mean value was recorded (in Kg) for analysis.

##### 2.5.2.3 Functional performance tests

Standing long jump: Participants stood with feet together behind a marked line, performed a countermovement, and jumped horizontally for maximal distance. Distance from the starting line to the nearest heel contact point was measured to the nearest centimeter. Two trials were performed with 1-minute rest intervals, and the longest distance (in meter) was recorded for analysis.

100-meter sprint: Sprint performance was assessed on a level outdoor track surface. Time to complete 100 meters from a standing start to crossing the finish line was recorded using electronic timing watch (accuracy ±0.01 seconds). A single trial was performed at each assessment time point.

Exercise-specific endurance outcomes: Participants performed different functional assessments based on baseline upper-body pulling capacity. Individuals able to safely perform straight-arm pull-ups completed repetitions from a standard overhead bar (pronated grip) until volitional exhaustion, with valid repetitions requiring full arm extension at the bottom position and the chin clearing the bar at the top. Participants unable to safely execute pull-ups performed a timed abdominal curl test, completing as many repetitions as possible within 60 seconds with knees flexed to 90°, hands positioned behind the head, and elbows required to contact the knees for each repetition to be counted as valid. Allocation to assessment type coincided with sex due to baseline strength differences but was determined by functional capability rather than sex itself.

##### 2.5.2.4 Safety monitoring and adverse event reporting

Participants were monitored continuously throughout all training sessions for signs of adverse events, including excessive pain, numbness, tingling, skin discoloration, or other symptoms suggestive of inadequate tissue perfusion or nerve compression. The attending physician (A.Z., Y.Y., I.A) was present at all sessions and empowered to immediately terminate exercise and remove cuffs if safety concerns arise.

Post-session interviews were conducted after the first, mid and final training sessions to systematically capture participant-reported symptoms addressing muscle soreness, fatigue, numbness, and overall tolerability.

### 2.6 Statistical analysis

Statistical analyses were conducted using IBM SPSS Statistics version 26.0 (IBM Corp., Armonk, NY, USA). Data normality was evaluated using the Shapiro–Wilk test in conjunction with visual inspection of box and QQ plots. Baseline demographic and anthropometric characteristics were compared between groups for continuous variables and chi-square tests for categorical variables.

Primary analyses used a two-way repeated-measures ANOVA with one within-subject factor (Time: baseline, post-intervention) and one between-subject factor (Group: BFR vs control) to test the main effects of Time and Group and, critically, the Time × Group interaction.

Where appropriate, *post hoc* comparisons were used to describe within-group pre–post changes and between-group differences at each time point; these were considered descriptive and interpreted in light of the interaction effect. Assumptions (normality of residuals and homogeneity of variances) were assessed; with two time points, sphericity is not applicable.

Subgroup analyses by gender were exploratory in nature. Formal statistical tests for interaction between group and gender were not performed due to limited statistical power. Instead, group means and standard deviations were examined and reported to identify potential gender-specific response patterns that may warrant further investigation in future studies.

Statistical significance was set at an alpha level of 0.05 (two-tailed) for all analyses. All analyses followed the intention-to-treat principle; as no outcome data were missing, ITT and complete-case analyses were identical.

## 3 Results

### 3.1 Participant characteristics

Forty-eight participants were enrolled and randomized to either the Low-load multi-component training with BFR group (n=24) or the Low-load multi-component training without BFR group (n=24). Baseline characteristics are presented in **Table 2**. No significant differences were observed between groups for any demographic variable. Age was comparable between the BFR group (19.04 ± 0.70 years) and the without-BFR group (18.92 ± 0.58 years; P = 0.50). Height did not differ between groups (BFR: 171.38 ± 5.11 cm vs. without-BFR: 170.12 ± 7.85 cm; P = 0.91), nor did weight (BFR: 64.33 ± 7.07 kg vs. without-BFR: 63.62 ± 9.17 kg; P = 0.59). Gender distribution was balanced, with 13 males (54.2%) in the BFR group and 12 males (50%) in the without-BFR group (P = 0.77). All participants were physically inactive university-aged adults. All 48 participants completed the full 6-week intervention. Compliance was defined as *a priori* as attendance at ≥85% of the prescribed exercise sessions (20/24), and all participants met or exceeded this threshold.

**Table 2:** Demographic characteristics of all participants that complete the study.

Demographic characteristics		BFR group (n = 24)	Control group (n = 24)	P
Age (years)		19.04 ± 0.70	18.92 ± 0.58	0.50 **^+^**
Height (cm)		171.38 ± 5.11	170.12 ± 7.85	0.91 **^+^**
Weight (kg)		64.33 ± 7.07	63.62 ± 9.17	0.59 **^+^**
Gender no (%)	Male	13 (54.2)	12 (50)	0.77^†^
	Female	11 (45.8)	12 (50)	
Education		Undergraduate	Undergraduate	

Values are represented as mean and standard deviation. BFR, indicates Blood Flow Restricted; ^+^, indicates Mann-Whitney U-Test; ^†^, Fisher exact test; P < 0.05 significant.

### 3.2 Muscle thickness assessed by musculoskeletal ultrasound imaging

Repeated measures ANOVA revealed significant main effects of time for all muscle thickness measurements, indicating that both groups experienced increases in muscle thickness following the intervention (**Table 3**). However, the presence of significant Time × Group interactions at several sites indicated that the magnitude of hypertrophic adaptation differed between the BFR and control groups.

**Table 3:** Result of muscle thickness following musculoskeletal ultrasound assessment with repeated measure ANOVA.

Group	Time	Primary outcome measure							
		BMTR	BMTL	RFMTR	RFMTL	GMTR	GMTL	SMTR	SMTL
BFR Group (n=24)	T1	1.82 ± 0.28	1.87 ± 0.24	1.57 ± 0.21	1.55 ± 0.28	1.51 ± 0.34	1.50 ± 0.32	1.61 ± 0.25	1.60 ± 0.26
	T2	2.27 ± 0.60	2.24 ± 0.45	1.70 ± 0.23	1.65 ± 0.26	1.59 ± 0.30	1.59 ± 0.35	1.70 ± 0.29	1.64 ± 0.26
Control Group(n=24)	T1	1.79 ± 0.51	1.76 ± 0.51	1.51 ± 0.23	1.50 ± 0.24	1.49 ± 0.36	1.47 ± 0.35	1.61 ± 0.27	1.52 ± 0.31
	T2	1.90 ± 0.52	1.86 ± 0.57	1.53 ± 0.23	1.52 ± 0.24	1.54 ± 0.34	1.52 ± 0.32	1.63 ± 0.26	1.60 ± 0.21
P (ηp^2^)	Time	< 0.001	< 0.001	< 0.001	0.006	< 0.001	< 0.001	< 0.001	0.005
	Time*Group	0.001	<0.001	0.004	0.04	0.33	0.07	0.01	0.35
	Group	0.14	0.06	0.09	0.21	0.72	0.61	0.64	0.40

Values are represented as mean and standard deviation. BMTR, Bicep Muscle thickness Right; BMTL, Bicep Muscle thickness Left; RFMTR, Rectus femoris Muscle thickness Right; RFMTL, Rectus femoris Muscle thickness left; GMTR, Gastrocnemius Muscle thickness Right; GMTL, Gastrocnemius Muscle thickness Left; SMTR, Soleus Muscle thickness Right; SMTL, Soleus Muscle thickness Left; ηp^2^ partial eta squared; P value calculated using Repeated measure ANOVA; P < 0.05 is significant.

For biceps brachii muscle thickness, repeated measures ANOVA demonstrated significant main effects of time for both the right (P < 0.001) and left (P < 0.001) sides, indicating increases in muscle thickness across both groups. Significant Time × Group interactions were observed for both right (P = 0.001) and left (P < 0.001) biceps, indicating greater increases in the BFR group compared to without BFR group. No significant main effect of group was found for the right (P = 0.14) or left (P = 0.06) biceps, confirming the absence of baseline differences between groups.

**Figure 4:**
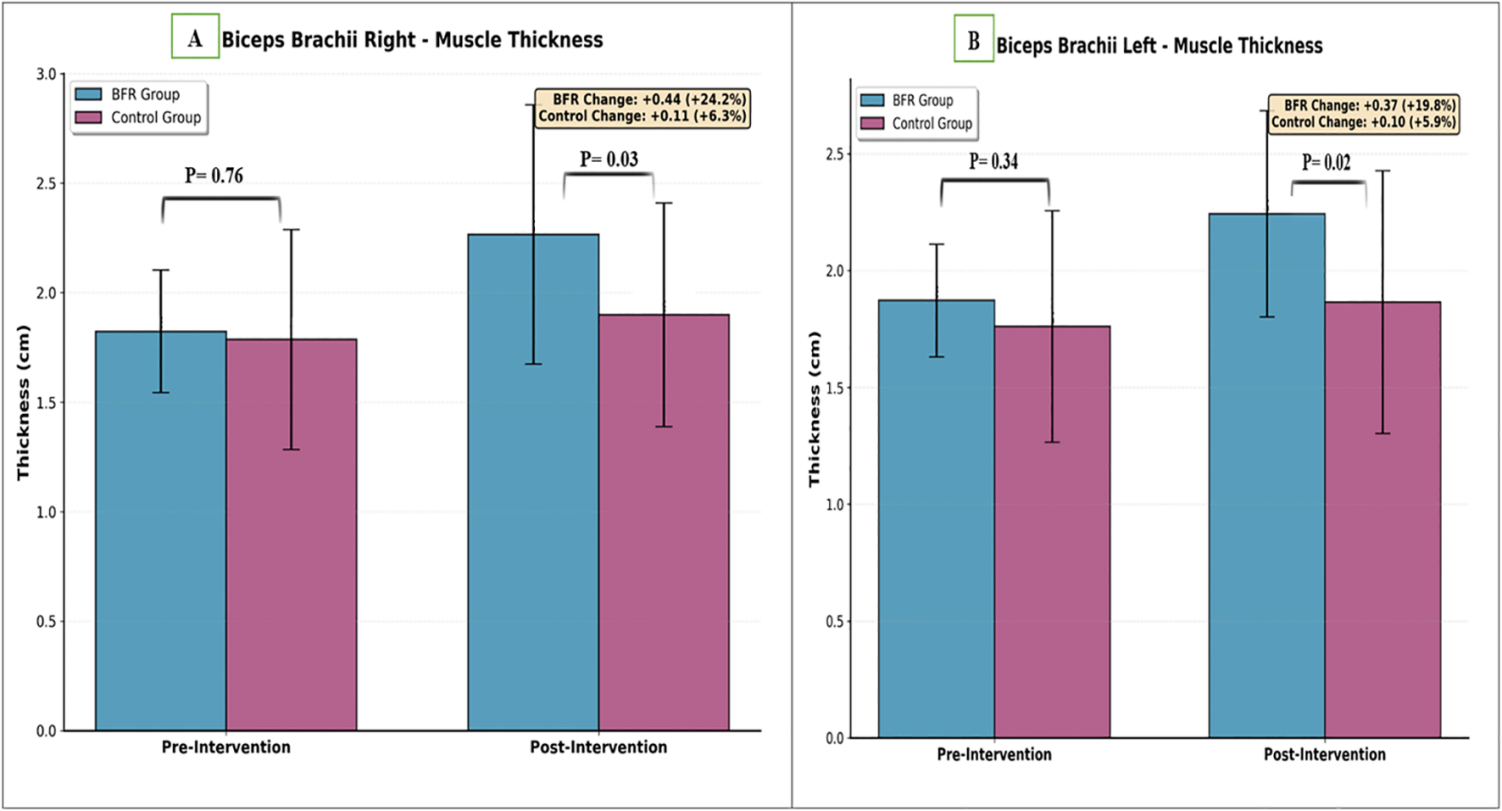
Comparison of bicep brachii muscle thickness between groups. **(A)** Right side, **(B)** Left side.

In the BFR group, right biceps thickness increased from 1.82 ± 0.28 cm at baseline to 2.27 ± 0.60 cm post-intervention, representing an absolute change of 0.45 cm. Left biceps thickness increased from 1.87 ± 0.24 cm to 2.24 ± 0.45 cm, an increase of 0.37 cm. In without-BFR group, right biceps thickness increased from 1.79 ± 0.51 cm to 1.90 ± 0.52 cm (change: 0.11 cm), while left biceps thickness increased from 1.76 ± 0.51 cm to 1.86 ± 0.57 cm (change: 0.10 cm) (**Table 3**; **Figures 4A, B**).

**Figure 5:**
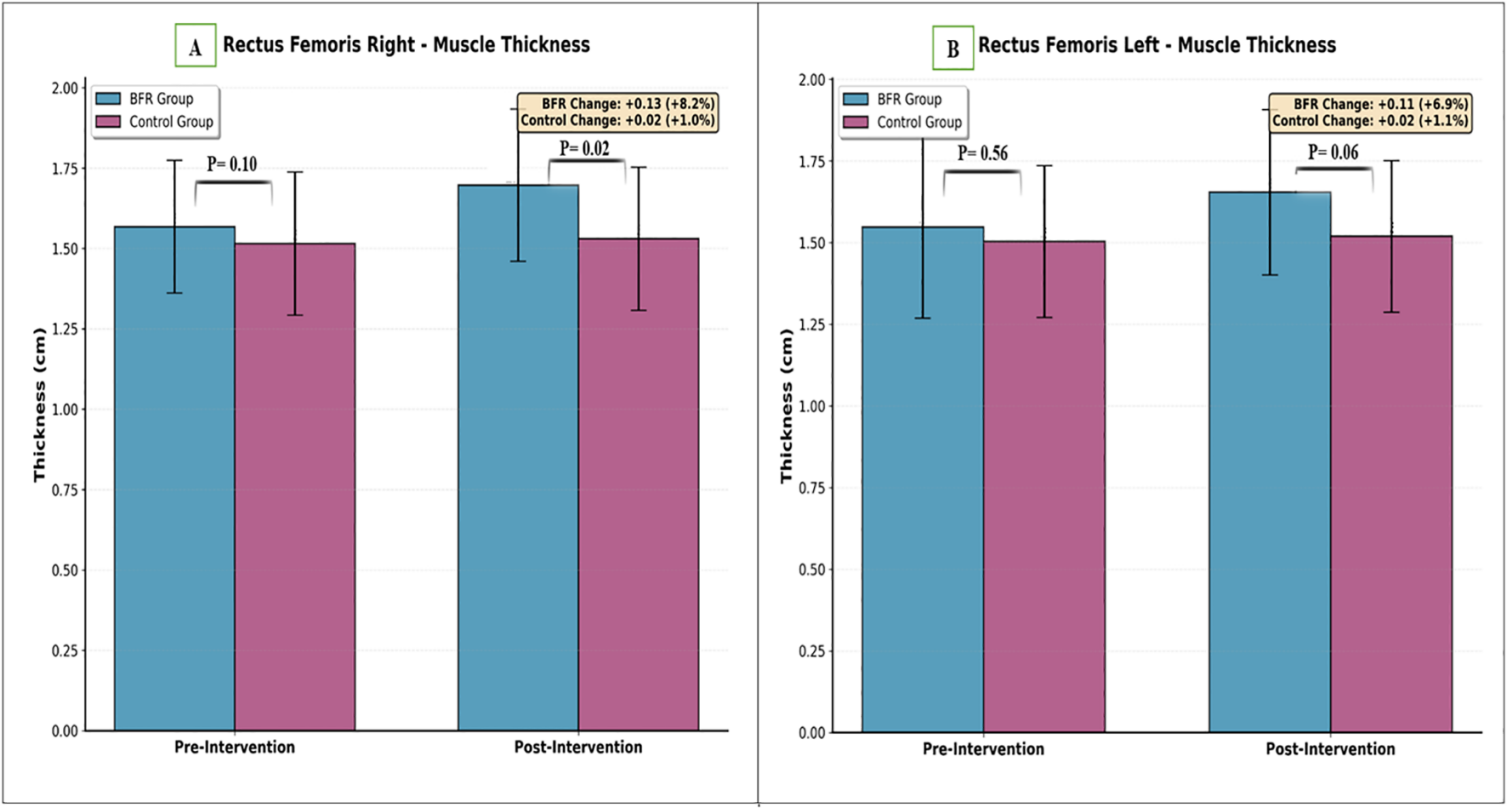
Comparison of rectus femoris muscle thickness between groups. **(A)** Right side, **(B)** Left side.

Repeated measures ANOVA revealed significant main effects of time for both right (P < 0.001) and left (P = 0.006) rectus femoris muscle thickness. Significant Time × Group interactions were observed for the right (P = 0.004) and left (P = 0.04) sides, indicating differential responses between groups. No baseline differences were detected between groups (right: P = 0.09; left: P = 0.21). In the BFR group, right rectus femoris thickness increased from 1.57 ± 0.21 cm to 1.70 ± 0.23 cm (change: 0.13 cm), and left rectus femoris thickness increased from 1.55 ± 0.28 cm to 1.65 ± 0.26 cm (change: 0.10 cm). In without-BFR group, right rectus femoris thickness increased from 1.51 ± 0.23 cm to 1.53 ± 0.23 cm (change: 0.02 cm), and left rectus femoris thickness increased from 1.50 ± 0.24 cm to 1.52 ± 0.24 cm (change: 0.02 cm) (**Table 3**; **Figures 5A, B**).

**Figure 6:**
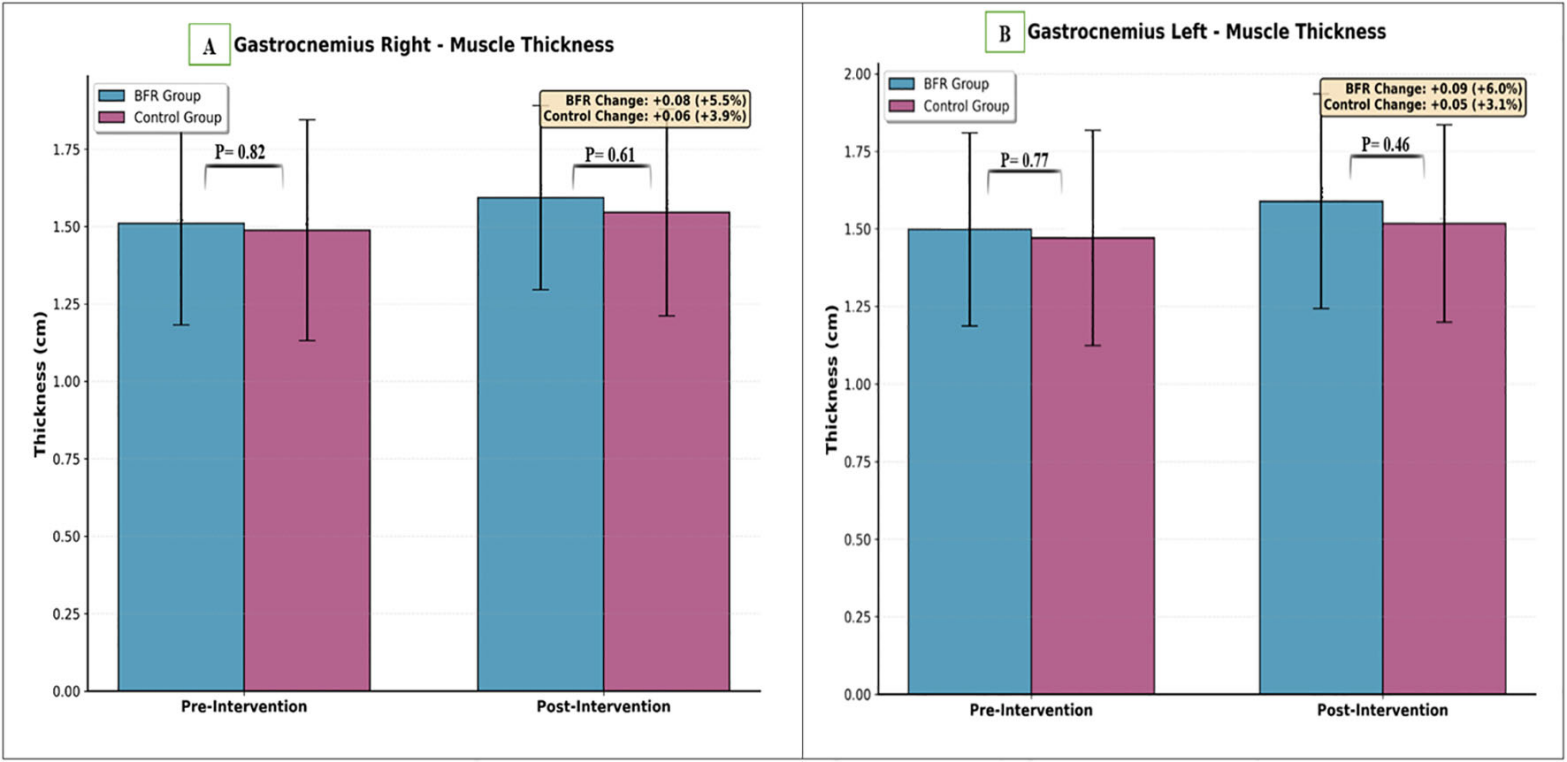
Comparison of gastrocnemius muscle thickness between groups. **(A)** Right side, **(B)** Left side.

Significant main effects of time were observed for both right (P < 0.001) and left (P < 0.001) gastrocnemius muscle thickness. However, the Time × Group interactions were not significant (right: P = 0.33; left: P = 0.07), indicating comparable increases in gastrocnemius thickness between groups. No baseline differences existed between groups (right: P = 0.72; left: P = 0.61). Right gastrocnemius thickness increased from 1.51 ± 0.34 cm to 1.59 ± 0.30 cm in the BFR group (change: 0.08 cm) and from 1.49 ± 0.36 cm to 1.54 ± 0.34 cm in without-BFR group (change: 0.05 cm). Left gastrocnemius thickness increased from 1.50 ± 0.32 cm to 1.59 ± 0.35 cm in the BFR group (change: 0.09 cm) and from 1.47 ± 0.35 cm to 1.52 ± 0.32 cm in without-BFR group (change: 0.05 cm) (**Table 3**; **Figures 6A, B**).

**Figure 7:**
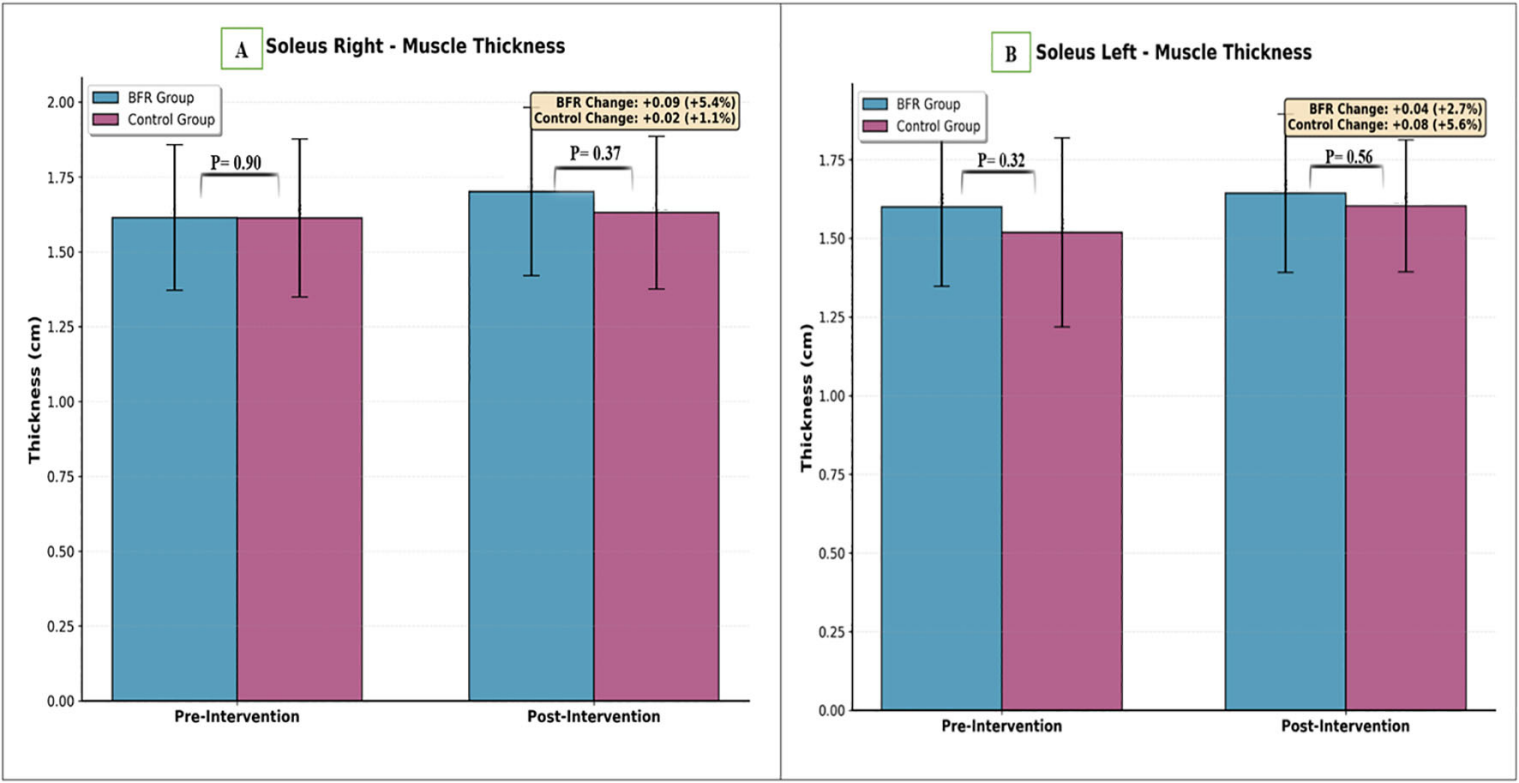
Comparison of soleus muscle thickness between groups. **(A)** Right side, **(B)** Left side.

Repeated measures ANOVA showed significant main effects of time for both right (P < 0.001) and left (P = 0.005) soleus muscle thickness. A significant Time × Group interaction was found for the right soleus (P = 0.01), but not for the left soleus (P = 0.35). No baseline group differences were detected (right: P = 0.64; left: P = 0.40). In the BFR group, right soleus thickness increased from 1.61 ± 0.25 cm to 1.70 ± 0.29 cm (change: 0.09 cm), and left soleus thickness increased from 1.60 ± 0.26 cm to 1.64 ± 0.26 cm (change: 0.04 cm). In without-BFR group, right soleus thickness increased from 1.61 ± 0.27 cm to 1.63 ± 0.26 cm (change: 0.02 cm), and left soleus thickness increased from 1.52 ± 0.31 cm to 1.60 ± 0.21 cm (change: 0.08 cm) (**Table 3**; **Figures 7A, B**).

### 3.3 Functional performance and anthropometric outcomes

Both groups showed considerable enhancements in functional performance and anthropometric parameters, with relatively greater changes in the BFR group for most outcomes. (**Supplementary Figures 9–13**).

#### 3.3.1 Grip strength and limb circumference

A significant main effect of time was found for both left (P < 0.001) and right (P < 0.001) handgrip strength. A significant Time × Group interaction was observed for left handgrip strength (P < 0.001), indicating greater improvements in the BFR group, but not for right handgrip strength (P = 0.14). No baseline differences were present for left (P = 0.17) or right (P = 0.08) handgrip strength. Left handgrip strength increased from 25.83 ± 3.94 kg to 29.46 ± 4.51 kg in the BFR group (change: 3.63 kg) and from 24.83 ± 6.95 kg to 25.92 ± 6.96 kg in without-BFR group (change: 1.09 kg). Right handgrip strength increased from 25.83 ± 3.26 kg to 28.38 ± 3.26 kg in the BFR group (change: 2.55 kg) and from 24.67 ± 3.49 kg to 26.33 ± 3.08 kg in without-BFR group (change: 1.66 kg) (**Table 4**; **Supplementary Figures 9A, B**).

**Table 4:** Result of physical performance measures with repeated measure ANOVA.

Group	Time	Primary outcome measure									
		GSL	GSR	CMUAL	CMUAR	CTL	CTR	SLJ	100mS	Pull-ups	Abdominal curls
BFR Group (n=24)	T1	25.83 ± 3.94	25.83 ± 3.26	27.75 ± 4.13	27.50 ± 4.22	52.58 ± 6.0	52.67 ± 5.73	2.13 ± 0.24	18.54 ± 1.61	8.77 ± 4.40	35.64 ± 6.99
	T2	29.46 ± 4.51	28.38 ± 3.26	29.96 ± 5.58	29.92 ± 4.90	55.61 ± 5.60	55.85 ± 5.38	2.29 ± 0.28	17.50 ± 1.71	14.69 ± 5.45	46.82 ± 8.29
Control Group (n=24)	T1	24.83 ± 6.95	24.67 ± 3.49	26.37 ± 3.03	26.08 ± 2.46	52.04 ± 3.05	52.12 ± 3.40	2.01 ± .029	19.38 ± 3.09	9.17 ± 3.66	35.25 ± 9.89
	T2	25.92 ± 6.96	26.33 ± 3.08	27.24 ± 3.05	27.38 ± 2.62	52.79 ± 3.23	53.07 ± 3.23	2.10 ± 0.32	18.85 ± 2.70	11.25 ± 3.02	38.58 ± 10.5
P	Time	< 0.001	< 0.001	< 0.001	< 0.001	< 0.001	< 0.001	< 0.001	< 0.001	< 0.001	< 0.001
	Time*Group	< 0.001	0.14	0.09	0.19	0.01	0.01	0.20	0.24	< 0.001	< 0.001
	Group	0.17	0.08	0.07	0.05	0.19	0.19	0.04	0.09	0.36	0.26

Data presented in mean ± standard deviation. GSL, Grip strength left; GSR, Grip strength right; CMUAL, Circumference of mid-upper arm left; CMUAR, Circumference of mid-upper arm right; CTL, Circumference of thigh left; CTR, Circumference of thigh right; SLJ, Standing long jump; AC, Abdominal curl females only; PU, Pull-up males only; 100mS, 100-meter sprint; P < 0.05 is significant. Grip strength in kg, Circumference in (cm), SLJ in meters, 100mS in seconds, Pull-ups and abdominal curl in number of repetitions.

Significant main effects of time were observed for both left (P < 0.001) and right (P < 0.001) mid-upper arm circumference. However, Time × Group interactions were not significant (left: P = 0.09; right: P = 0.19), indicating that increases in arm circumference were similar between groups. No baseline differences were detected (left: P = 0.07; right: P = 0.05). Left mid-upper arm circumference increased from 27.75 ± 4.13 cm to 29.96 ± 5.58 cm in the BFR group (change: 2.21 cm) and from 26.37 ± 3.03 cm to 27.24 ± 3.05 cm in the control group (change: 0.87 cm). Right mid-upper arm circumference increased from 27.50 ± 4.22 cm to 29.92 ± 4.90 cm in the BFR group (change: 2.42 cm) and from 26.08 ± 2.46 cm to 27.38 ± 2.62 cm in without-BFR group (change: 1.30 cm) (**Table 4**; **Supplementary Figures 10A, B**).

Repeated measures ANOVA revealed significant main effects of time for both left (P < 0.001) and right (P < 0.001) thigh circumference. Significant Time × Group interactions were found for both the left (P = 0.01) and right (P = 0.01) thigh, indicating greater increases in the BFR group. No baseline differences existed between groups (left: P = 0.19; right: P = 0.19). Left thigh circumference increased from 52.58 ± 6.0 cm to 55.61 ± 5.60 cm in the BFR group (change: 3.03 cm) and from 52.04 ± 3.05 cm to 52.79 ± 3.23 cm in without-BFR group (change: 0.75 cm). Right thigh circumference increased from 52.67 ± 5.73 cm to 55.85 ± 5.38 cm in the BFR group (change: 3.18 cm) and from 52.12 ± 3.40 cm to 53.07 ± 3.23 cm in without-BFR group (change: 0.95 cm) (**Table 4**; **Supplementary Figures 11A, B**).

#### 3.3.2 Power and speed performance

A significant main effect of time was observed (P < 0.001), indicating improvements in both groups. However, the Time × Group interaction was not significant (P = 0.20), suggesting comparable improvements between groups. A significant main effect of group was detected (P = 0.04), but this reflected a baseline difference, with the BFR group demonstrating greater jump distance at baseline. Standing long jump distance increased from 2.13 ± 0.24 m to 2.29 ± 0.28 m in the BFR group (change: 0.16 m) and from 2.01 ± 0.29 m to 2.10 ± 0.32 m in without-BFR group (change: 0.09 m) (**Table 4**; **Supplementary Figure 12**).

A significant main effect of time was found (P < 0.001), indicating improvements in sprint performance in both groups. The Time × Group interaction was not significant (P = 0.24), suggesting similar improvements between groups. No baseline difference was observed (P = 0.09). Sprint time decreased from 18.54 ± 1.61 s to 17.50 ± 1.71 s in the BFR group (change: −1.04 s) and from 19.38 ± 3.09 s to 18.85 ± 2.70 s in without-BFR group (change: −0.53 s) (**Table 4**; **Supplementary Figure 13**).

### 3.4 Exercise-specific muscular endurance performance

Repeated measures ANOVA revealed significant Time × Group interactions for both pull-up performance (P < 0.001) and abdominal curl performance (P < 0.001), indicating greater improvements in the BFR group compared with the without-BFR group for each exercise modality. No baseline differences were observed between groups for pull-ups (P = 0.36) or abdominal curls (P = 0.26).

**Figure 8:**
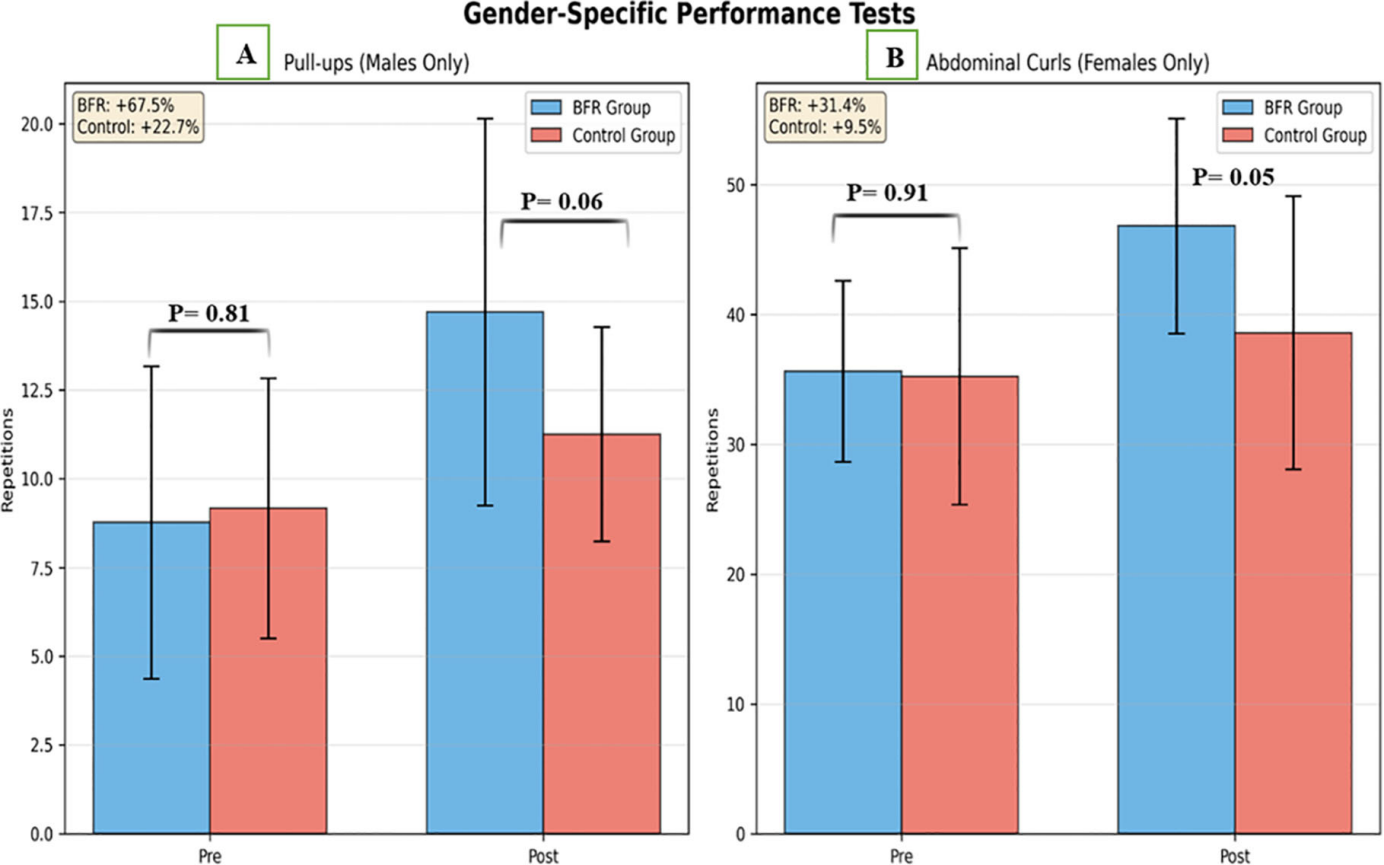
Comparison of gender specific performance between groups. **(A)** Pull-Up (males only), **(B)** Abdominal Curl (Females Only).

Among participants who performed the pull-up assessment, repetitions increased from 8.77 ± 4.40 to 14.69 ± 5.45 in the BFR group (change: +5.92 repetitions) and from 9.17 ± 3.66 to 11.25 ± 3.02 in the without-BFR group (change: +2.08 repetitions) (**Figure 8A**). Among participants who completed the abdominal curl assessment, repetitions increased from 35.64 ± 6.99 to 46.82 ± 8.29 in the BFR group (change: +11.18 repetitions) and from 35.25 ± 9.89 to 38.58 ± 10.53 in the without-BFR group (change: +3.33 repetitions) (**Figure 8B**). Because different functional tests were used based on baseline capacity, these findings should be interpreted as exercise-specific adaptations rather than sex-based differences.

#### 3.4.1 Male participants

In the male subgroup (BFR n = 13, control n = 12), the BFR group demonstrated greater improvements compared to without-BFR group across several outcome measures (**Table 5**). Upper extremity changes included increases in right biceps thickness (BFR: +0.63 cm, +32.7%; without-BFR: +0.05 cm, +2.5%) and left biceps thickness (BFR: +21.2%; without-BFR: +9.8%), as well as improvements in left handgrip strength (BFR: +16.2%; without-BFR: +8.6%).

**Table 5:** Gender-specific improvement in both groups.

Outcome measure	BFR males change (n=13)	BFR females change (n=11)	Control males change (n=12)	Control females change (n=12)
MSK Ultrasound				
BMTR	+0.63 (+32·7%)	+0.22 (+13·0%)	+0.05 (+2·5%)	+0.18 (+10.8%)
BMTL	+0.42 (+21·2%)	+0.32 (+18·0%)	+0.18 (+9·8%)	+0.03 (+1·6%)
RFMTR	+0.16 (+9·7%)	+0.10 (+6·4%)	+0.01 (+0·4%)	+0.03 (+1·6%)
RFMTL	+0.08 (+5·2%)	+0.13 (+9·0%)	+0.01 (+0·6%)	+0.02 (+1·6%)
GMTR	+0.08 (+5·3%)	+0.09 (+5·8%)	+0.07 (+4·7%)	+0.04 (+3·0%)
GMTL	+0.11 (+7·6%)	+0.06 (+4·2%)	+0.02 (+1·5%)	+0.07 (+4·9%)
SMTR	+0.09 (+5·1%)	+0.09 (+5·8%)	+0.01 (+0·7%)	+0.02 (+1·5%)
SMTL	+0.02 (+1·3%)	+0.07 (+4·6%)	+0.06 (+3·6%)	+0.11 (+8·0%)
Functional performance and anthropometric outcomes				
GSL	+4.38 (+16·2%)	+2.73 (+11·2%)	+1.83 (+8·6%)	+0.33 (+1·2%)
GSR	+2.92 (+10·7%)	+2.09 (+8·7%)	+3.33 (+14·3%)	+0.00 (+0·0%)
CMUAL	+1.58 (+6·1%)	+2.95 (+9·8%)	+1.42 (+5·5%)	+0.32 (+1·2%)
CMUAR	+2.08 (+8·3%)	+2.82 (+9·3%)	+2.50 (+9·6%)	+0.08 (+0·3%)
CTL	+4.05 (+8·1%)	+1.82 (+3·3%)	+1.08 (+2·1%)	+0.42 (+0·8%)
CTR	+3.77 (+7·6%)	+2.50 (+4·4%)	+1.69 (+3·3%)	+0.20 (+0·4%)
SLJ	+0.19 (+8·6%)	+0.12 (+5·9%)	+0.12 (+5·4%)	+0.07 (+3·6%)
100mS	−1.49 (−8·1%)	−0.52 (−2·8%)	−0.54 (−2·9%)	−0.51 (−2·5%)

Values are presented in mean (percentage). BMTR, Bicep Muscle thickness Right; BMTL, Bicep Muscle thickness Left; RFMTR, Rectus femoris Muscle thickness Right; RFMTL, Rectus femoris Muscle thickness left; GMTR, Gastrocnemius Muscle thickness Right; GMTL, Gastrocnemius Muscle thickness Left; SMTR, Soleus Muscle thickness Right; SMTL, Soleus Muscle thickness Left; GSL, Grip strength left; GSR, Grip strength right; CMUAL, Circumference of mid-upper arm left; CMUAR, Circumference of mid-upper arm right; CTL, Circumference of thigh left; CTR, Circumference of thigh right; SLJ, Standing long jump; 100mS, 100-meter sprint.

Lower extremity changes included increases in right rectus femoris thickness (BFR: +9.7%; without-BFR: +0.4%), left gastrocnemius thickness (BFR: +7.6%; without-BFR: +1.5%), and bilateral thigh circumference (left: BFR + 8.1% vs. without-BFR +2.1%; right: BFR + 7.6% vs. without-BFR +3.3%).

#### 3.4.2 Female participants

In the female subgroup (BFR n = 11, control n = 12), the BFR group showed greater improvements compared with the control group across multiple outcomes (**Table 5**). Upper extremity changes included increases in left biceps thickness (BFR: +0.32 cm, +18.0%; without-BFR: +1.6%), bilateral handgrip strength (left: BFR + 11.2% vs. without-BFR +1.2%; right: BFR + 8.7% vs. without-BFR +0.0%), and bilateral mid-upper arm circumference (left: BFR + 9.8% vs. without-BFR +1.2%; right: BFR + 9.3% vs. without-BFR +0.3%).

Lower extremity changes included increases in left rectus femoris thickness (BFR: +9.0%; without-BFR: +1.6%) and bilateral thigh circumference (left: BFR + 3.3% vs. without-BFR +0.8%; right: BFR + 4.4% vs. without-BFR +0.4%).

### 3.5 Adverse events

No significant adverse events were documented during the 6-week intervention. Seven participants (29%) in the BFR group experienced minor transient discomfort during the first week of training, including light muscle soreness, brief numbness, or tingling. All symptoms resolved spontaneously without intervention. No participant withdrew from the study due to adverse events.

## 4 Discussion

This randomized controlled trial examined whether the addition of blood flow restriction (BFR) to a standardized low-load multi-component training program would enhance muscle hypertrophy and functional performance outcomes compared with the same program performed without BFR in physically inactive young adults. The BFR group demonstrated significantly greater improvements in bilateral biceps thickness, right rectus femoris thickness, left-hand grip strength, bilateral thigh circumference, and exercise-specific muscular endurance performance (pull-ups in males, abdominal curls in females), representing 10 of 18 between-group comparisons (56%). These findings indicate that, when implemented with appropriate safety considerations, the addition of BFR to low-load multi-component training may provide additional hypertrophic and functional benefits beyond the same program performed without BFR.

### 4.1 Principal findings in context of low-load training

Our findings contribute to the growing body of evidence supporting BFR as a method to augment adaptations from low-load resistance training. Previous research has established that low-load resistance training alone (typically <50% 1-RM) produces modest hypertrophic responses in untrained individuals, particularly when volume is sufficient (Loenneke et al., 2012b). The present study extends this literature by demonstrating that the addition of BFR to low-load training (~30–40% 1-RM) resulted in greater muscle thickness increases compared with matched low-load training without BFR, particularly in the upper extremities.

A meta-analysis by Lixandrão et al., 2018. reported that low-load training with BFR produced hypertrophic responses comparable to high-load training (≥65% 1-RM) in some contexts, though the present study did not include a high-load comparator and therefore cannot directly address this comparison. More relevant to our design, Loenneke et al (Loenneke et al., 2012b). demonstrated in their meta-analysis that low-load BFR training produced greater muscle strength and hypertrophy than low-load training alone, which aligns with our observed between-group differences in muscle thickness. However, our findings should not be interpreted as demonstrating superiority of BFR over high-load resistance training for maximal strength development, as we did not assess directly the 1-RM strength and did not include a high-load training group (Lixandrão et al., 2018) (Wang et al., 2025).

Ma et al., 2024 conducted a systematic review examining BFR combined with resistance training in young adults, reporting that BFR interventions produced muscle strength improvements comparable to traditional resistance training. While our results support the efficacy of low-load training with BFR, our functional performance measures (grip strength, pull-ups, abdominal curls) assess different constructs than maximal strength (1-RM testing) and should be interpreted accordingly. The observed improvements in functional performance likely reflect a combination of hypertrophic, neuromuscular, and task-specific adaptations rather than purely maximal force-generating capacity.

### 4.2 Hypertrophic adaptations: magnitude and distribution

The muscle thickness increases observed in the BFR group were most pronounced in the biceps brachii (right: +24.7%; left: +19.8%) and right rectus femoris (+8.3%), with more modest changes in the gastrocnemius and soleus muscles. This regional variation in hypertrophic response may reflect differences in the metabolic stress experienced during the specific exercises performed. Upper extremity exercises in our protocol (bicep curls, push-ups) may have induced greater metabolic accumulation under BFR conditions compared with lower extremity exercises, potentially due to differences in muscle mass recruited, exercise duration, or occlusion effectiveness at different anatomical sites (Kataoka, [[NoYear]]).

The observed biceps hypertrophy in our study (+24.7% over 6 weeks in the BFR group) is within the upper range of responses reported in previous short-term BFR interventions. Nancekievill et al (Nancekievill et al., 2025). reported significant increases in lean body mass following 6 weeks of BFR training in both males and females using dual-energy X-ray absorptiometry (DXA), though direct comparisons are limited by different assessment methods. Our use of musculoskeletal ultrasound provided site-specific measurements, allowing identification of regional response patterns that whole-body composition methods may not detect.

The more modest improvements in lower extremity muscle thickness, particularly in the gastrocnemius and soleus, may reflect several factors. First, these muscles play key roles in habitual locomotion and postural tasks (e.g., during stance and walking), which may influence their responsiveness to a short-duration, low-load intervention (Hamilton et al., 2022) (Lenhart et al., 2014) (Lai et al., 2015). Second, the multi-component nature of the program (sprinting/plyometrics plus resistance/bodyweight exercises) distributed training volume across multiple muscle groups and performance qualities, which may reduce the specificity of stimulus for any single muscle compared with a more targeted plantarflexor hypertrophy protocol (Fyfe et al., 2016) (Fyfe et al., 2014). Third, the effective BFR stimulus can vary as a function of cuff pressure, limb characteristics, and exercise conditions; therefore, local anatomical factors and exercise selection may have influenced the degree of restriction-related stress experienced by the posterior lower leg musculature in our protocol (Patterson et al., 2019) (Bartolomei et al., 2022).

### 4.3 Functional performance improvements and underlying mechanisms

The BFR group demonstrated greater improvements than the without-BFR group in several functional performance measures, including left-hand grip strength (+14.1% vs +4.4%), bilateral thigh circumference (+5.8–6.0% vs +1.4–1.8%), and exercise-specific muscular endurance performance. Importantly, these improvements should be interpreted as enhancements in task-specific functional capacity rather than direct indicators of maximal strength, as we did not assess directly 1-RM performance.

The mechanisms underlying functional performance improvements following BFR training are likely to extend beyond simple muscle hypertrophy. While increased muscle cross-sectional area typically contributes to force production capacity, several additional factors may have contributed to the observed functional gains. First, neuromuscular adaptations, including improved motor unit recruitment patterns and firing rates, have been demonstrated during BFR exercise (Iskarevskii et al., 2024) (Olmos et al., 2024). Olmos et al. reported that BFR increased motor unit firing rates during moderate-load muscle actions, suggesting enhanced neural drive that could improve performance independent of hypertrophic changes (Olmos et al., 2024).

Second, the pronounced metabolic stress associated with BFR may promote hypertrophic adaptations with a substantial non-contractile (sarcoplasmic) component (Curovic, 2025). Short-term, low-load BFR interventions may induce increases in sarcoplasmic volume (glycogen, water, non-contractile proteins) that contribute to muscle thickness and circumference measurements but do not proportionally increase maximal force production capacity. This distinction is relevant when interpreting the functional significance of the observed morphological changes, particularly in the absence of maximal strength testing.

Third, task-specific adaptations related to the training exercises themselves likely contributed to improved performance on functionally similar assessments (Vigouroux and Devise, 2024) (Lee et al., 2021) (Stone et al., 2022). For example, the multi-component program included upper-body pulling, abdominal curl and grip-demanding patterns, which would be expected to improve performance on pull-up and curl tests and grip dynamometry through skill acquisition and movement pattern optimization, independent of hypertrophy.

### 4.4 Potential physiological mechanisms

Although the present study did not include mechanistic measurements, the observed adaptations may be contextualized within existing mechanistic literature on BFR training. It should be emphasized that the following discussion is speculative and based on previous research rather than direct measurements from this trial.

The restricted venous return during BFR exercise creates a localized hypoxic and metabolically stressful environment characterized by accumulation of metabolic byproducts (lactate, inorganic phosphate, hydrogen ions) (Lavigne et al., 2025) (Hayat et al., 2025) (Curovic, 2025). This metabolic stress is hypothesized to be a primary driver of muscle adaptation during BFR training. Koloskov et al., 2025 identified metabolic stress as the principal mechanism driving muscle growth during BFR exercise, proposing that metabolite accumulation induces muscle cell edema, which acts as a stimulus for hypertrophy. The cell swelling hypothesis proposes that the accumulation of metabolites and fluid within muscle cells may activate mechanosensitive pathways, including the mammalian target of rapamycin (mTOR) signaling cascade, a central regulator of protein synthesisn (Li et al., 2025) (He et al., 2025). While conceptually plausible, it should be noted that we did not measure mTOR activation, muscle protein synthesis rates, or intracellular signaling, and therefore cannot confirm that these pathways mediated the observed adaptations in our study.

BFR may influence motor unit recruitment patterns during low-load exercise. Iskarevskii et al., 2024 reported that BFR affected motor unit recruitment thresholds and amplitude-frequency characteristics during exercise, potentially allowing low-load exercise to recruit higher-threshold motor units typically activated only during high-load contractions. Such altered recruitment patterns could contribute to both hypertrophic and functional adaptations, though we did not assess neuromuscular function in the present study.

Acute hormonal responses to BFR exercise, including elevations in growth hormone (GH) and insulin-like growth factor-1 (IGF-1), have been reported in some studies (Yinghao et al., 2021) (Aram et al., 2024). However, the physiological significance of transient post-exercise hormone elevations for muscle hypertrophy remains controversial, and we did not measure hormonal responses in this trial. While systemic hormonal responses may contribute to the whole-body adaptations observed, definitive conclusions regarding their role cannot be drawn from the present data.

### 4.5 Gender-specific responses and exercise-specific adaptations

The gender-stratified analyses revealed that males in the BFR group demonstrated greater improvements than male without-BFR in 12 outcome measures, while females in the BFR group showed advantages in 13 measures. However, several methodological considerations limit the interpretation of these findings as sex-specific physiological responses.

First, muscular endurance outcomes were assessed using different tests in males (pull-ups) and females (abdominal curl-ups) based on baseline feasibility and safety. Because these tasks evaluate distinct anatomical regions and performance constructs, upper-body pulling performance versus trunk endurance, they are not directly comparable across sexes. Accordingly, the observed improvements should be interpreted as exercise-specific responses to adding BFR within the low-load program (i.e., greater gains in pull-up repetitions among those tested with pull-ups and greater gains in abdominal curl repetitions among those tested with curl-ups), rather than evidence that males and females differ in physiological responsiveness to BFR. Moreover, the present study was not powered to test a formal Group × Time × Sex interaction, and mechanistic inferences (e.g., fiber-type–specific or sex-dependent adaptation pathways) cannot be drawn from these exercise-specific outcomes. Future trials should include at least one common functional test administered to all participants and be powered for interaction analyses to evaluate whether BFR effects differ by sex.

BFR training showed substantial advantages for both genders, however with variations in response patterns. Previous research also has provided mixed evidence regarding sex differences in BFR responses. Nancekievill et al., 2025 reported similar relative increases in lean body mass between males and females following 6 weeks of BFR training, though males demonstrated somewhat greater strength improvements. Reece et al., 2023 observed similar whole-muscle hypertrophy between sexes but reported that males experienced greater myofiber-level hypertrophy, suggesting that sex-specific adaptations may occur at different organizational levels. Refalo et al., 2025 conducted a systematic review indicating that females can achieve muscle hypertrophy comparable to males when expressed as relative gains, supporting the efficacy of resistance training across sexes.

The practical implication of our gender-stratified findings is that both males and females demonstrated meaningful responses to low-load training with BFR across multiple outcomes, suggesting that BFR-enhanced training may be beneficial for both sexes. However, claims of sex-specific adaptation mechanisms or preferential responsiveness to BFR require more rigorous study designs, including appropriate statistical testing of sex interactions and controlled use of equivalent functional assessments.

### 4.6 Measurement considerations: ultrasound and anthropometry

Our use of B-mode musculoskeletal ultrasound for muscle thickness assessment provided site-specific quantification of morphological changes with minimal influence from subcutaneous adipose tissue or fluid retention (Högelin et al., 2022) (Lohmann et al., 2024). This methodology offers advantages over circumference measurements alone, which can be confounded by changes in subcutaneous tissue, edema, or measurement variability. The consistent patterns observed between ultrasound-derived muscle thickness and anthropometric circumference measurements (e.g., increased biceps thickness accompanied by increased mid-upper arm circumference) provide converging evidence for genuine muscle hypertrophy rather than transient fluid shifts.

However, ultrasound measurements also have limitations. Muscle thickness represents only one dimension of muscle size and may not fully capture changes in muscle length, pennation angle, or architectural properties that could influence functional capacity. Additionally, operator technique and probe pressure can influence measurement reliability, though we implemented standardized protocols and assessor training to minimize these sources of error (Högelin et al., 2022) (Lohmann et al., 2024).

### 4.7 Limitations

Several limitations should be considered. First, the intervention lasted only 6 weeks, so we cannot determine whether the observed benefits of adding BFR persist, plateau, or change over longer training periods. Second, participants were healthy, physically inactive young adults (18–20 years), which limits generalizability to trained individuals, older adults, or clinical populations. Third, we did not directly assess maximal strength (true 1-RM); therefore, our findings relate to muscle thickness and task-specific functional performance and should not be interpreted as superiority over high-load training for maximal strength development. Fourth, different endurance tests were used in males (pull-ups) and females (abdominal curl-ups), preventing direct sex comparisons; moreover, the study was not powered for a formal Group × Time × Sex interaction. Fifth, key lifestyle factors such as dietary protein intake, sleep, and habitual activity were not controlled, which may have contributed to variability in hypertrophic responses. Sixth, while the overall sample size (n=48) was adequate for the main analyses, it limited power for subgroup analyses and for exploring inter-individual variability. Seventh, given the number of outcomes assessed (n = 18), no correction for multiple comparisons was applied, therefore, the possibility that some between-group differences reached significance by chance cannot be excluded, and findings should be interpreted collectively. Finally, the study did not include a high-load comparator, so the relative magnitude of effects versus traditional high-load resistance training cannot be established.

### 4.8 Future directions

Future studies should build on these findings in several practical ways. First, longer trials (e.g., ≥12 weeks) are needed to determine whether the added benefits of BFR over low-load training alone are sustained over time and how best to progress BFR variables. Second, dose–response research should systematically test key prescription parameters (e.g., occlusion pressure, cuff width, volume, and frequency) and account for individual differences that may influence responsiveness. Third, mechanistic studies incorporating measures such as muscle oxygenation, neuromuscular assessments, and, where feasible, molecular or imaging markers could clarify how BFR drives adaptation and why some individuals respond more than others. Fourth, trials in broader populations (trained athletes, older adults, and clinical groups) (Feng et al., 2025) are needed to confirm generalizability and to strengthen safety evidence in higher-risk settings. Finally, comparative and pragmatic studies (including high-load comparators and real-world delivery in clinics, gyms, or home-based programs) should evaluate effectiveness, adherence, feasibility, and cost-effectiveness to guide implementation.

### 4.9 Practical implications

Adding BFR to a matched low-load multi-component training program produced modest improvements in muscle thickness and selected functional outcomes compared with the same program performed without BFR. This may be useful for people who cannot tolerate, or prefer to avoid, high-load resistance training (e.g., due to joint pain, prior injury, or fear of heavy lifting). In our physically inactive young adults, the approach was feasible and well tolerated, with no serious adverse events and only brief, minor discomfort reported mainly during the first week.

In rehabilitation settings, BFR may help preserve or build muscle when heavy loading is temporarily inappropriate (e.g., early after injury or surgery), but it should be implemented with appropriate contraindication screening and monitoring (Patterson et al., 2019) (Jørgensen et al., 2016) (Wang et al., 2022). BFR is best viewed as a complementary option rather than a replacement for high-load training, which remains the most established strategy for maximizing maximal strength in healthy individuals (American College of Sports Medicine, 2009) (Lixandrão et al., 2018) (Wang et al., 2025).

This work aligns with SDG 3 by supporting scalable strategies to improve fitness in inactive young adults, with links to SDG 4 (evidence-based education in exercise/rehabilitation) and SDG 10 (reducing access barriers to health-promoting activity) (Feng et al., 2025).

## 5 Conclusions

Low-load multi-component training program with BFR improved muscle thickness and selected functional outcomes compared with matched low-load multi-component training program without-BFR; however, prior evidence indicates that high-load resistance training typically produces greater maximal strength gains than low-load BFR, so BFR should be viewed primarily as a practical low-load alternative when heavy loading is not feasible or desired. In this randomized controlled trial, low-load multi-component training with BFR resulted in greater improvements in upper limb muscle thickness and certain functional outcomes than similar training without BFR, although effects varied across outcomes in young adults. BFR was well tolerated with no serious adverse events, and future research should examine long-term adherence, durability of adaptations, optimal prescription parameters, and generalizability to older, clinical, and athletic populations.

## Data Availability

The raw data supporting the conclusions of this article will be made available by the authors, without undue reservation.
