# Supplementary material for: Effects of a low-load multi-component training program with blood flow restriction versus the same program without blood flow restriction on muscle thickness and functional outcomes in physically inactive young adults: randomized controlled trial
Source: Front Physiol. 2026 Apr 1;17:1792481. doi: 10.3389/fphys.2026.1792481 (PMC13079173; doi:10.3389/fphys.2026.1792481)
Supplement: Supplementary file 1 [file DataSheet1.pdf]

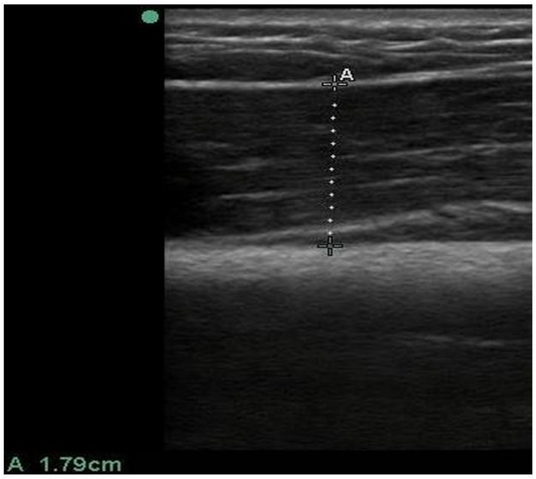

3Ba. Bicep Brachii muscle

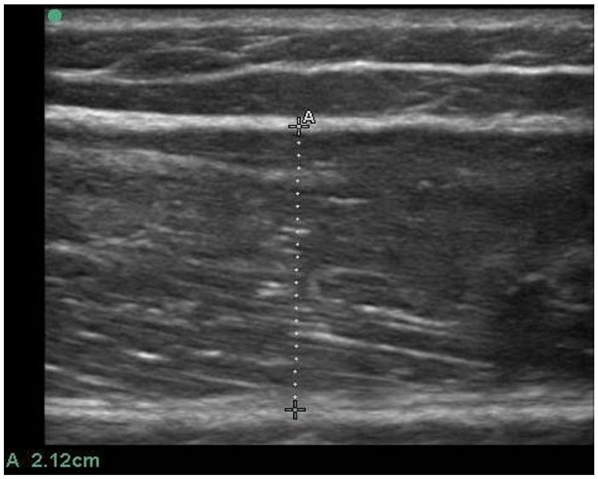

3Bb. Rectus Femoris muscle

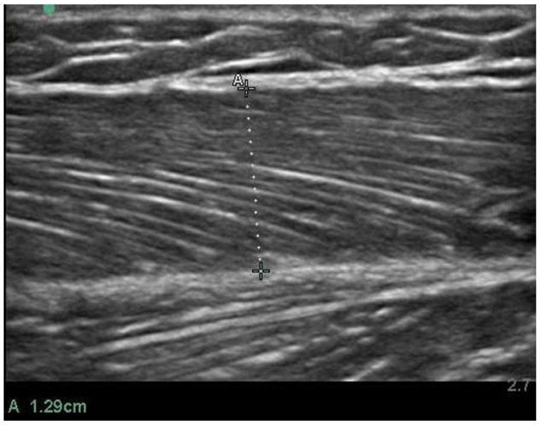

3Bc. Gastrocnemius muscle

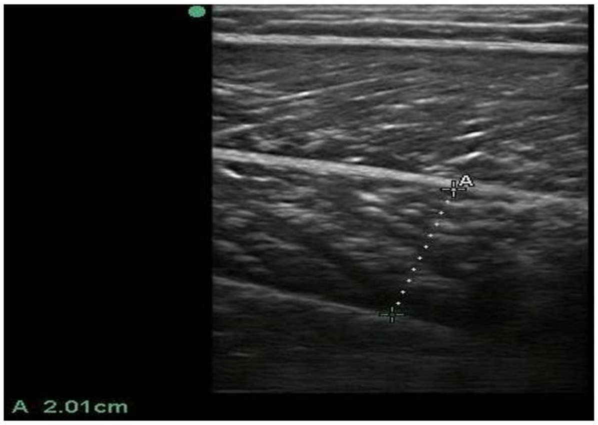

3Bd. Soleus muscle

Supplementary Figure 3B. Ultrasound Images of Upper and lower limb muscles.

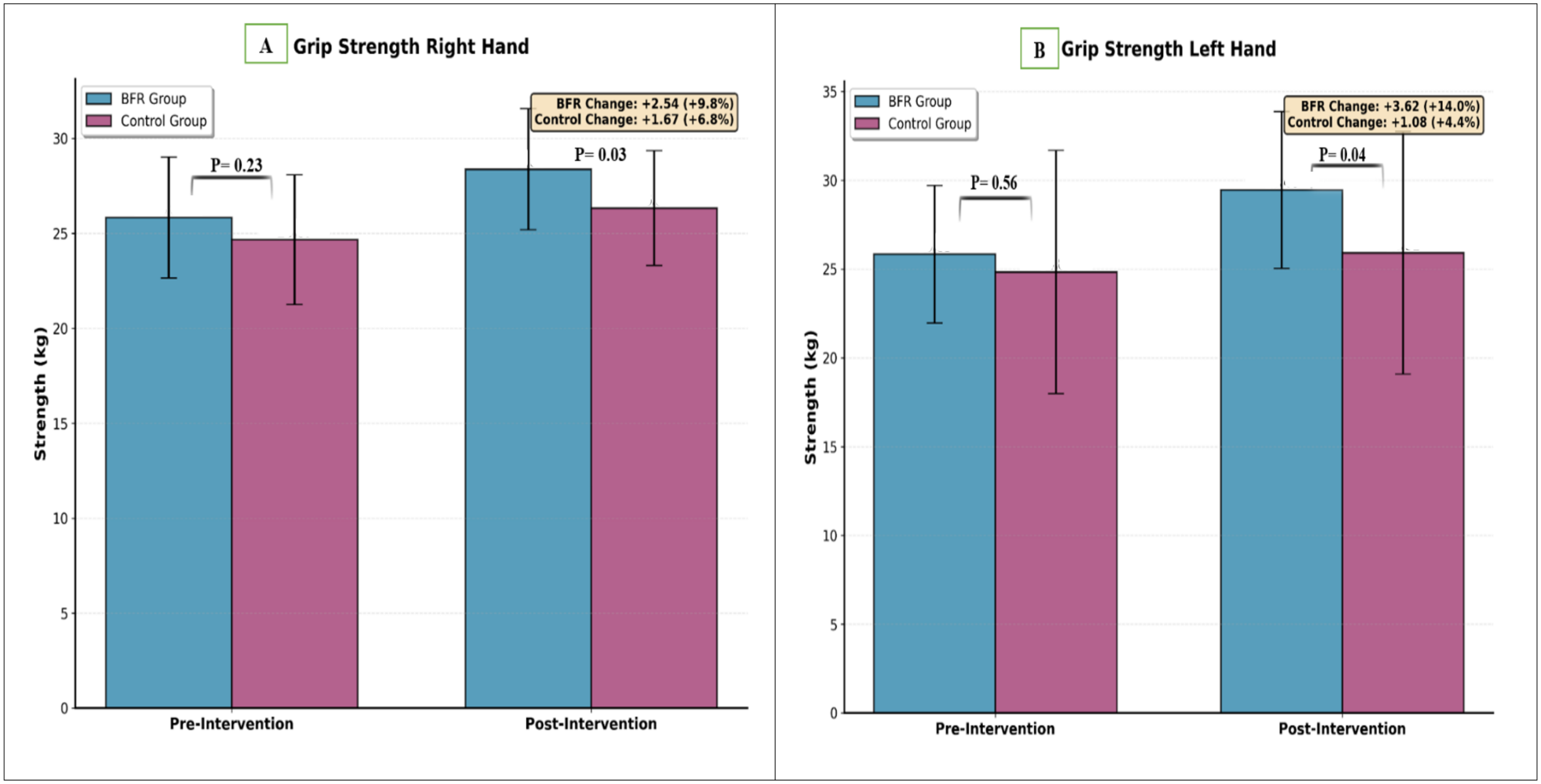

Supplementary Figure 9: Comparison of hand grip strength between Groups. A) Right side, B) Left side

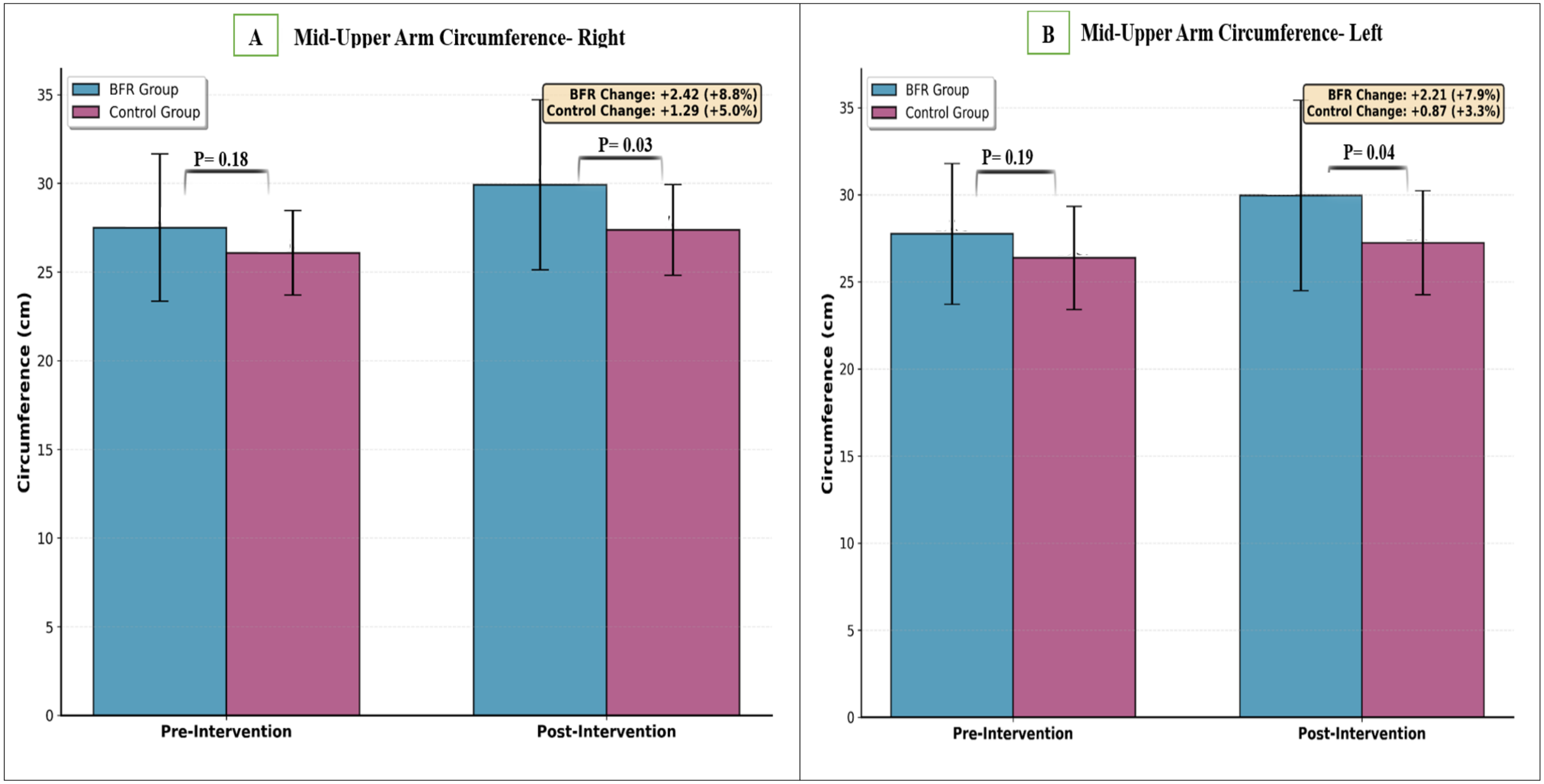

Supplementary Figure 10: Comparison of Mid-Upper arm circumference between Groups. A) Right side, B) Left side

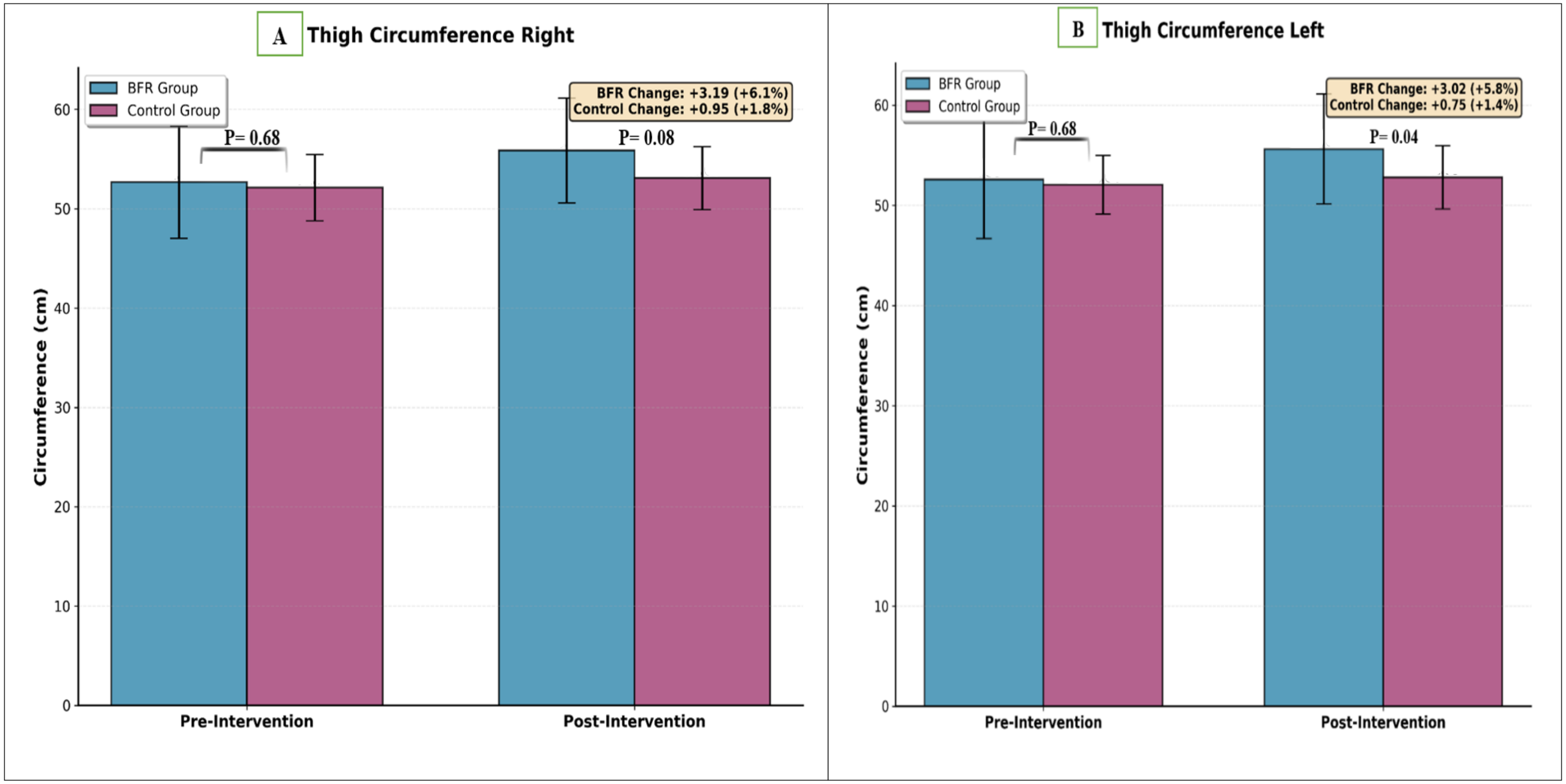

Supplementary Figure 11: Comparison of thigh circumference between Groups. A) Right side, B) Left side

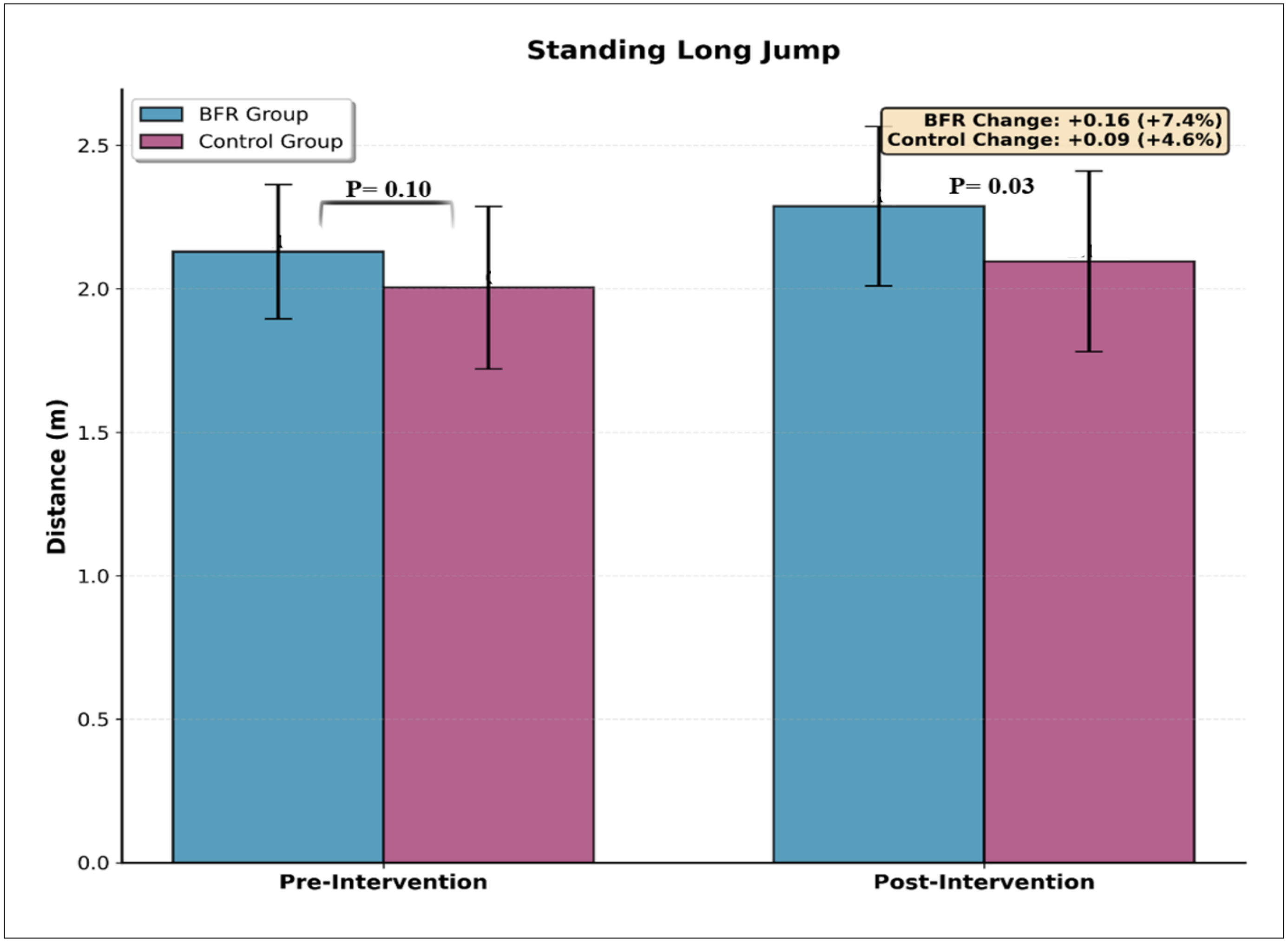

Supplementary Figure 12: Comparison of Standing Long Jump between Groups

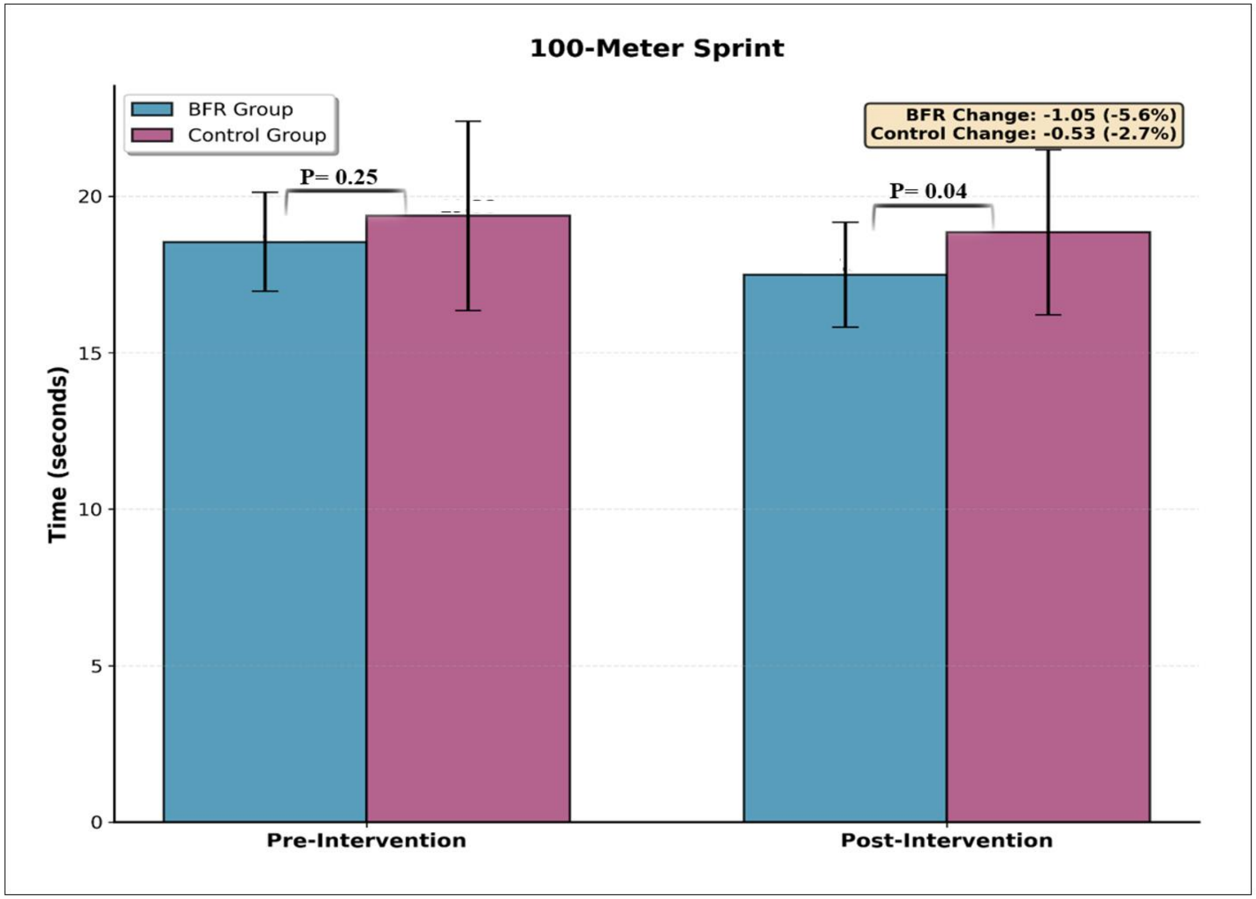

Supplementary Figure 13: Comparison of 100- Meter Sprint between Groups
